# Functional Characterization of a Novel Class of Morantel-Sensitive Acetylcholine Receptors in Nematodes

**DOI:** 10.1371/journal.ppat.1005267

**Published:** 2015-12-01

**Authors:** Elise Courtot, Claude L. Charvet, Robin N. Beech, Abdallah Harmache, Adrian J. Wolstenholme, Lindy Holden-Dye, Vincent O’Connor, Nicolas Peineau, Debra J. Woods, Cedric Neveu

**Affiliations:** 1 INRA, UMR1282 Infectiologie et Santé Publique, Nouzilly, France; 2 Université de François Rabelais de Tours, UMR1282 Infectiologie et Santé Publique, Tours, France; 3 Institute of Parasitology, McGill University, Macdonald Campus, Sainte Anne de Bellevue, Québec, Canada; 4 Department of Infectious Disease and Center for Tropical and Emerging Global Disease, University of Georgia, Athens, Georgia, United States of America; 5 School of Biological Sciences, University of Southampton, Southampton, United Kingdom; 6 Université François Rabelais de Tours, Département de physiologie animale, Tours, France; 7 Veterinary Medicine Research and Development, Zoetis LLC, Kalamazoo, Michigan, United States of America; University of Massachusetts Medical School, UNITED STATES

## Abstract

Acetylcholine receptors are pentameric ligand–gated channels involved in excitatory neuro-transmission in both vertebrates and invertebrates. In nematodes, they represent major targets for cholinergic agonist or antagonist anthelmintic drugs. Despite the large diversity of acetylcholine-receptor subunit genes present in nematodes, only a few receptor subtypes have been characterized so far. Interestingly, parasitic nematodes affecting human or animal health possess two closely related members of this gene family, *acr-26* and *acr-27* that are essentially absent in free-living or plant parasitic species. Using the pathogenic parasitic nematode of ruminants, *Haemonchus contortus*, as a model, we found that Hco-ACR-26 and Hco-ACR-27 are co-expressed in body muscle cells. We demonstrated that co-expression of Hco-ACR-26 and Hco-ACR-27 in *Xenopus laevis* oocytes led to the functional expression of an acetylcholine-receptor highly sensitive to the anthelmintics morantel and pyrantel. Importantly we also reported that ACR-26 and ACR-27, from the distantly related parasitic nematode of horses, *Parascaris equorum*, also formed a functional acetylcholine-receptor highly sensitive to these two drugs. In *Caenorhabditis elegans*, a free-living model nematode, we demonstrated that heterologous expression of the *H*. *contortus* and *P*. *equorum* receptors drastically increased its sensitivity to morantel and pyrantel, mirroring the pharmacological properties observed in *Xenopus* oocytes. Our results are the first to describe significant molecular determinants of a novel class of nematode body wall muscle AChR.

## Author Summary

The control of parasitic nematode infections in humans, livestock and companion animals is critically dependent on anthelmintic treatment. However, the indiscriminate use of anthelmintic drugs has inevitably led to the selection of resistant parasites. In this respect there is currently an urgent need to increase our knowledge of the mode of action of available anthelmintics as well as to identify novel targets for the development of next generation anthelmintic compounds. In the present study we report the functional and pharmacological characterization of a novel subtype of nematode acetylcholine-gated ion channel in two distantly related parasitic nematode species: *Haemonchus contortus* and *Parascaris equorum*. Using the *Xenopus laevis* oocyte as an expression system, we showed that these receptors are composed of subunits encoded by two closely related genes, *acr-26* and *acr-27* that are widely distributed in parasitic nematodes infecting humans and animals. We further demonstrate that these receptors represent a molecular target for the anthelmintics morantel and pyrantel. The *H*. *contortus* and *P*. *equorum* receptors expressed as transgenes in the nematode model *Caenorhabditis elegans* both confer morantel and pyrantel sensitivity *in-vivo*. For parasitic nematodes of veterinary and medical importance, this novel class of cholinergic receptor is of prime interest for target-based drug screening and the development of relevant anthelmintic combinations.

## Introduction

Parasitic nematodes have a major impact on both human and animal health worldwide. In the absence of an efficient alternative strategy such as vaccination, the control of these parasites relies mainly on three major classes of anthelmintic compounds including the benzimidazoles, the macrocyclic lactones and the agonists or antagonists of ligand-gated acetylcholine receptors.

However, during the past 50 years, both the widespread and indiscriminate use of the available anthelmintics has led to the selection of resistant parasites. This is currently a major concern for the husbandry of small ruminants where highly pathogenic species such as *Haemonchus contortus* have developed resistance to the three major anthelmintic families [1, 2]. Anthelmintic resistance is also widespread in horse parasitic nematodes, such as *Parascaris equorum* and cyathostomins [3] and to a lesser extent, in dog hook-worm and heartworm [4–7]. Importantly, anthelmintic treatment failures have also been reported for both gastro-intestinal and filarial nematodes [8–12]. Therefore, there is an urgent need for a better understanding of anthelmintic mode of action and identification of novel anthelmintic targets to control resistant parasites and optimize drug application strategies [13].

The cholinergic system of parasitic nematodes has proven to be an efficient pharmacological target for anthelmintics [14, 15]. Cholinergic agonists such as levamisole, pyrantel and oxantel selectively open ligand-gated acetylcholine ion channels (AChRs) expressed in nematode body wall muscles to induce contraction of muscle cells leading to a spastic paralysis of the worms [16–20].

The AChRs are a pentameric assembly of five subunits that are designated as α- or non-α based on the presence of a cysteine doublet in their amino-acid sequence. Some α-subunits have the ability to associate together to create functional homopentameric receptors whereas non-α subunits have to associate with α-subunits in order to generate functional heteropentameric receptors.

Even though nematodes possess a large diversity of both α- and non-α AChR subunits (at least 29 in the model nematode *Caenorhabditis elegans*) only a small number of potential AChR subunit combinations have been characterized [21]. The *Xenopus laevis* oocyte has proven to be an efficient heterologous expression system to predict native nematode AChR subunit composition and define their pharmacological properties [22–30]. In addition, the heterologous expression of parasitic nematode AChRs has allowed the identification of the likely determinants that define potential molecular targets for anthelmintic compounds such as levamisole [25, 27], derquantel [30], tribendimidine [30] and monepantel [31]. Strikingly, depending on the nematode species under investigation, these studies highlighted some major differences in their subunit composition and their pharmacological properties. For example, whereas the functional expression of the *C*. *elegans* levamisole-sensitive AChR (L-AChR) requires the co-expression of five distinct subunits (UNC-38, UNC-63, LEV-8, LEV-1 and UNC-29) [24], we previously reported that, for the closely related trichostrongylid species *H*. *contortus* and *Oesophagostumum dentatum*, a combination of UNC-38, UNC-63; UNC-29 and ACR-8 subunits was sufficient to form functional L-AChRs [25, 30]. In this respect, even though *C*. *elegans* data provides an invaluable basis to decipher AChR from parasitic nematodes, the AChR diversity and specificity remain to be further explored in parasitic species.

The recent completion of genome and transcriptome sequencing from many nematode species has opened the way for the identification of new drug targets including a subset of AChR subunits that are specifically present in nematode parasites [28, 32]. Functional AChR subtypes containing such subunits represent potential pharmacological targets of prime interest for the development of novel parasite-selective anthelmintics. Accordingly, Bennett *et al*. recently reported the identification of ACR-26, an AChR α-subunit widely distributed in human and animal parasitic nematodes but absent in several free-living species and plant parasitic nematodes [28]. However, failure to obtain reliable and reproducible expression of homomeric ACR-26 channels from *Ascaris suum* or *H*. *contortus* in *Xenopus* oocytes suggests the requirement for additional subunits or chaperones to form functional AChRs.

In the present study we report the characterization of ACR-27 a novel AChR non-α subunit that is closely related to ACR-26. We show that *acr-26* and *acr-27* genes are present in parasitic nematode species from Clade III, Clade IV and Clade V including the most pathogenic species impacting human or animal health but absent from a majority of plant parasitic and free living species. Using *H*. *contortus* (Clade V) as a model, we show that *Hco-acr-26* and *Hco-acr-27* are co-expressed in body wall muscle cells from different developmental stages of the parasite and that co-expression of Hco-ACR-26 and Hco-ACR-27 in *Xenopus* oocytes leads to robust expression of a functional heteropentameric channel (Hco-26/27). Moreover, we demonstrate that ACR-26 and ACR-27 subunits from the distantly related Clade III parasitic species, *Parascaris equorum*, are also able to form a functional AChR when expressed in *Xenopus* oocyte (Peq-26/27). This receptor has a similar pharmacology pattern to Hco-26/27. Both Hco-26/27 and Peq-26/27 were expressed as transgenes in the model nematode *C*. *elegans* confirming the pharmacological properties of ACR-26 and ACR-27 containing receptors and highlighting their potential as parasite drug targets.

## Results

### *acr-27* genes are closely related to a*cr-26* and are widely represented in nematode phyla

Using the *Haemonchus contortus acr-26* deduced amino-acid sequences as a query, tBLASTn searches against *H*. *contortus* genomic data available at the Sanger Institute (www.sanger.ac.uk) allowed the identification of a partial sequence encoding a distinct AChR subunit for which no clear homolog could be identified in *Caenorhabditis elegans*. The corresponding full-length cDNA (1446 bp) was obtained by RT-PCR experiments and was named *Hco-acr-27* following the nomenclature proposed by Beech *et al*. [33]. The *Hco-acr-27* sequence was deposited to Genbank under the accession number KC790461.

In the general NCBI non redundant protein database, Hco-ACR-27 homologs represented only nematode sequences whereas Hco-ACR-26 homologs were identified in nematodes and also in mollusc species such as *Lottia gigantean*, *Aplysia californica*, the annelid species *Capitella telata* and the arthropod species *Ixodes scapularis* and *Daphnia pulex*.

**Fig 1:**
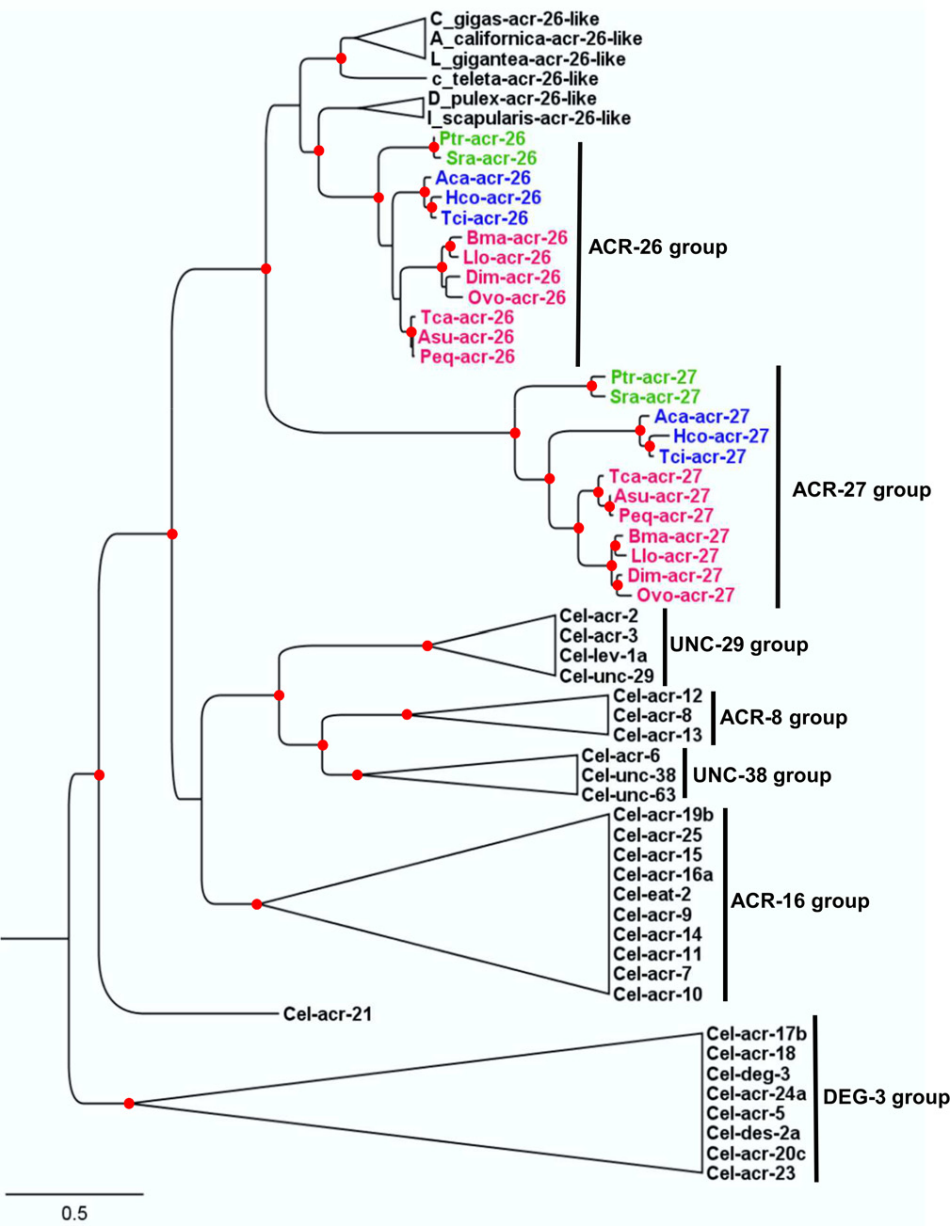
Maximum likelihood tree showing relationships of ACR-26 and ACR-27 acetylcholine receptor (AChR) subunits from parasitic nematodes with *C*. *elegans* AChR subunits. Tree was built upon an alignment of AChR subunit sequences excluding the predicted signal peptide and the highly variable region between TM3 and TM4. Potential homologs of ACR-26 identified in the molluscs, *Aplysia californica*, *Crassostrea gigas*, *Lottia gigantean*, the annelid *Capitella telata* and the arthropods *Ixodes scapularis* and *Daphnia pulex* were also included in the analysis. The tree was rooted with DEG-3 group subunit sequences. Branch lengths are proportional to the number of substitutions per amino acid. Scale bar represents the number of substitution per site. Red dots at the nodes indicate bootstrap values >70%. Accession numbers for sequences used in the phylogenetic analysis are provided in Materials and Methods section. *C*. *elegans* AChR subunit groups were named as proposed by Mongan *et al*. [35]. The three letter prefixes in AChR subunit gene names, *Cel*, *Tci*, *Hco*, *Aca*, *Tca*, *Peq*, *Ovo*, *Dim*, *Llo*, *Bma*, *Sra and Ptr* refer to *Caenorhabditis elegans*, *Teladorsagia circumcincta*, *Haemonchus contortus*, *Ancylostoma caninum*, *Toxocara canis*, *Parascaris equorum*, *Onchocerca volvulus*, *Dirofilaria immitis*, *Loa loa*, *Brugia malayi*, *Strongyloides ratti* and *Parastrogyloides trichosuri* respectively. Parasitic nematode species belonging to Clade III, VI and V are highlighted in red, green and blue respectively.

We were able to identify complete or partial *Hco-acr-26* and *Hco-acr-27* homolog in 44 and 42 nematode genomes respectively from a total of 88 genomes screened representing the four main clades of the phylum Nematoda (Clade I, III, IV and V) [34] (S1 Table). Strikingly, *acr-27* homologs were found to be specifically present in the nematode species for which an *acr-26* homolog could be identified. Our analysis revealed that *acr-26* and *acr-27* homologs were present within species belonging to Clade III, IV and V whereas no homolog of *acr-26* or *acr-27* could be identified in Clade I nematode species. Among the nematodes where both *acr-26* and *acr-27* homologs could be identified, a large majority (38) corresponded to parasitic species of mammals including species of the orders Ascaridida, Spirurida, and Strongylidida, which have an impact on human or animal health. Interestingly, as previously reported for *acr-26* [28], *acr-27* homologs were found to be essentially absent from plant parasitic and free-living species with two exceptions: *Rhabditophanes sp* KR3021 and *Panagrellus redivivus* that are free-living species from Clade IV. Also it is noteworthy that a potential *acr-26* homolog (but no *acr-27*) was found in the genome of the plant parasitic nematode *Bursaphelenchus xylophilus* (Clade IV). Fig 1 and S1 Fig show a phylogeny of *acr-26* and *acr-27* sequences from 12 parasitic nematode species representing Clade III, IV and V together with *acr-26* homologs identified in molluscs, arthropods and an annelid with cation channel subunit sequences from *C*. *elegans* for reference. The *acr-26*/*acr-27* clade is clearly distinct from the *acr-16*, *unc-38*, *unc-29* and *deg-3* clades previously identified [35]. The divergence of *acr-27* from *acr-26* suggests that *acr-27* arose from *acr-26* early in animal evolution prior to the divergence of protostomes and deuterostomes.

**Fig 2:**
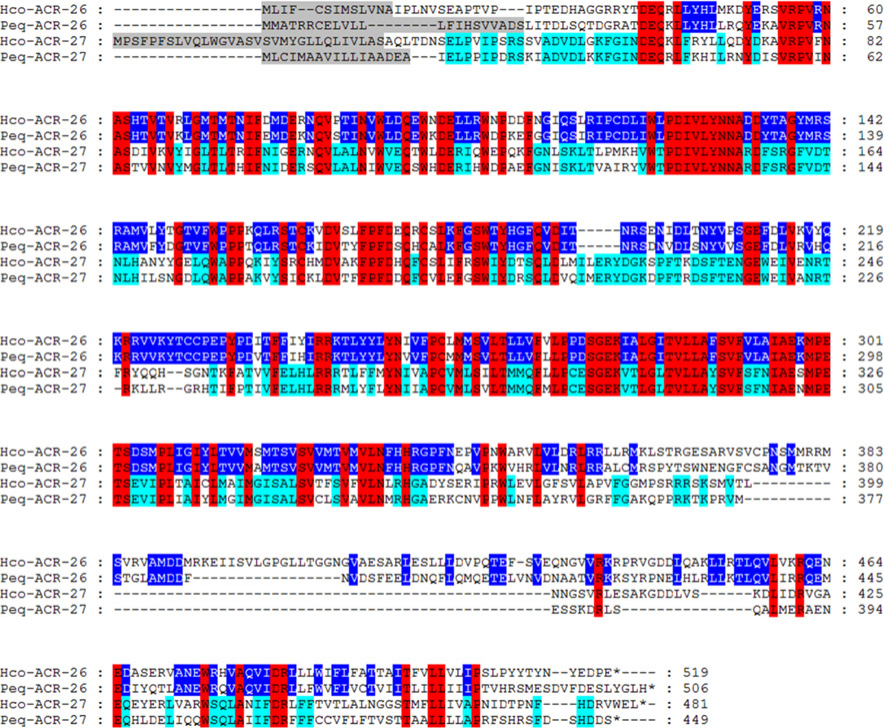
Amino-acid alignments of ACR-26 and ACR-27 AChR subunit sequences from *Haemonchus contortus* and *Parascaris equorum*. *acr-26* and *acr-27* deduced amino-acid sequences from *Haemonchus contortus* and *Parascaris equorum* were aligned using the MUSCLE algorithm [36] and further processed using GeneDoc. Predicted signal peptide sequences are shaded in grey. Amino acids conserved between ACR-26 and ACR-27 sequences are highlighted in red. Amino acids specifically shared by ACR-26 homologs are highlighted in dark blue. Amino acids specifically shared by ACR-27 homologs are highlighted in light blue. The cys-loop, the four transmembrane regions (TM1–TM4) and the primary agonist binding (YxCC) which is present in ACR-26 homologs but absent in ACR-27 homologs are noted above the sequences. Hco (*Haemonchus contortus*), Peq (*Parascaris equorum*).

A full-length cDNA from *acr-26* and *acr-27* was obtained from *Parascaris equorum*, a distantly related nematode species. An alignment of ACR-26 and ACR-27 subunit sequences from *H*. *contortus* (Clade V) and *P*. *equorum* (Clade III) is presented in Fig 2. All sequences shared typical features of an AChR subunit including a predicted signal peptide, a “cys-loop” and four transmembrane domains. Protein sequences were well conserved between the two nematode species with identities for the mature proteins, excluding the signal peptide sequence, ranging from 64% for ACR-27 to 69.7% for ACR-26. As previously reported, ACR-26 sequences possess the conserved vicinal cysteine motif (YxCC) in the potential acetylcholine-binding site defining them as α-subunits [28] whereas ACR-27 sequences lack this characteristic motif defining them as non-α-alpha subunits. This differential feature was confirmed in other nematode species belonging to Clade III, IV and V (S2 Fig).

The *acr-26* and *acr-27* clades cluster together distantly from other AChR subunits and since ACR-27 has characteristics of non-α subunits that must combine with α-subunits to form functional receptors [37], we raised the hypothesis that ACR-26 and ACR-27 subunits might associate to form a functional receptor.

### In *H*. *contortus*, *acr-26* and *acr-27* are expressed in the same developmental stages and the same tissues

**Fig 3:**
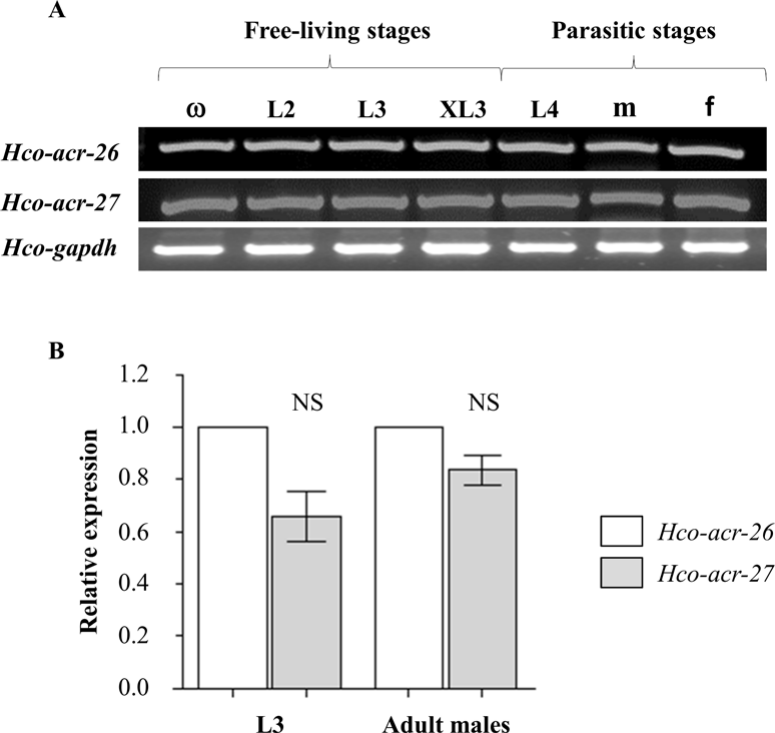
Expression monitoring of *Hco-acr-26* and *Hco-acr-27* in *Haemonchus contortus*. **(A)** Transcription of *Hco-acr-26* and *Hco-acr-27* throughout the *H*. *contortus* lifecycle as revealed by RT-PCR experiments. ω: embryonated egg; L2: second stage larvae; L3: ensheated third stage larvae; XL3: *in vitro* exsheated third stage larvae, L4: fourth stage larvae; m: adult males; f: adult females. Integrity of cDNA preparations was verified by PCR using primers designed to amplify a 380 bp fragment of the *H*. *contortus* gapdh cDNA. **(B)** Relative mRNA expression levels of *Hco-acr-26* and *Hco-acr-27* in *H*. *contortus* L3 and adult males. Real-time RT-PCR experiments were performed in triplicate for third stage larvae (L3) and adult males corresponding to free-living and parasitic stage respectively. Each set of experiments was repeated twice using two independent cDNAs templates. The mRNA expression level for *Hco-acr-26* was normalized to 1. The mRNA fold changes were calculated using three distinct reference genes encoding for gapdh, actin and β-tubulin. The data are presented as fold changes in mean ± SEM of mRNA expression. No difference was observed between relative mRNA expression of *Hco-acr-26* and *Hco-acr-27* in L3 and adult males (paired Student’s t-test).

**Fig 4:**
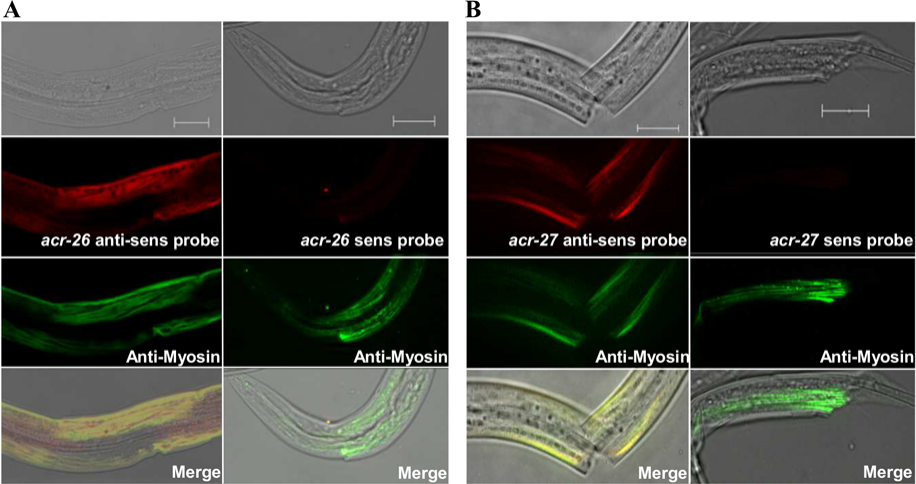
*In-situ* hybridizations of *Hco-acr-26* and *Hco-acr-27* mRNAs in *H*. *contortus*. XL3 larvae of *H*. *contortus* were fixed and hybridized with digoxygenin labeled antisense cDNA probes to monitor the localization of *Hco-acr-26* **(panel A)** and *Hco-acr-27* mRNAs **(panel B)** within the worm. Sense probes were used as negative control. cDNA/mRNA hybridomes were detected using primary anti-digoxygenin antibodies in combination with secondary Alexa 594-labeled antibodies (red). Body wall muscular cells were stained using primary antibodies raised against the myosin protein and further revealed with secondary Alexa 488-labeled antibodies (green). The scale bars correspond to 20 μm.

Transcripts of *Hco-acr-26* and *Hco-acr-27* were detected in all developmental stages including free-living embryonated eggs, L2 larvae, infective L3 larvae, *in-vitro* ex-sheathed L3 larvae (XL3) and parasitic L4 larvae (5dpi) and adult males and females 21 dpi, (Fig 3A). Both genes were expressed at similar levels in *H*. *contortus* L3 and adult males (Fig 3B). For both *Hco-acr-26* and *Hco-acr-27* mRNAs, *in-situ* hybridization experiments performed on the *H*. *contortus* XL3 stage led to a robust and reproducible labeling overlapping with anti-myosin immuno-staining, providing evidence of their muscular expression (Fig 4).

**Fig 5:**
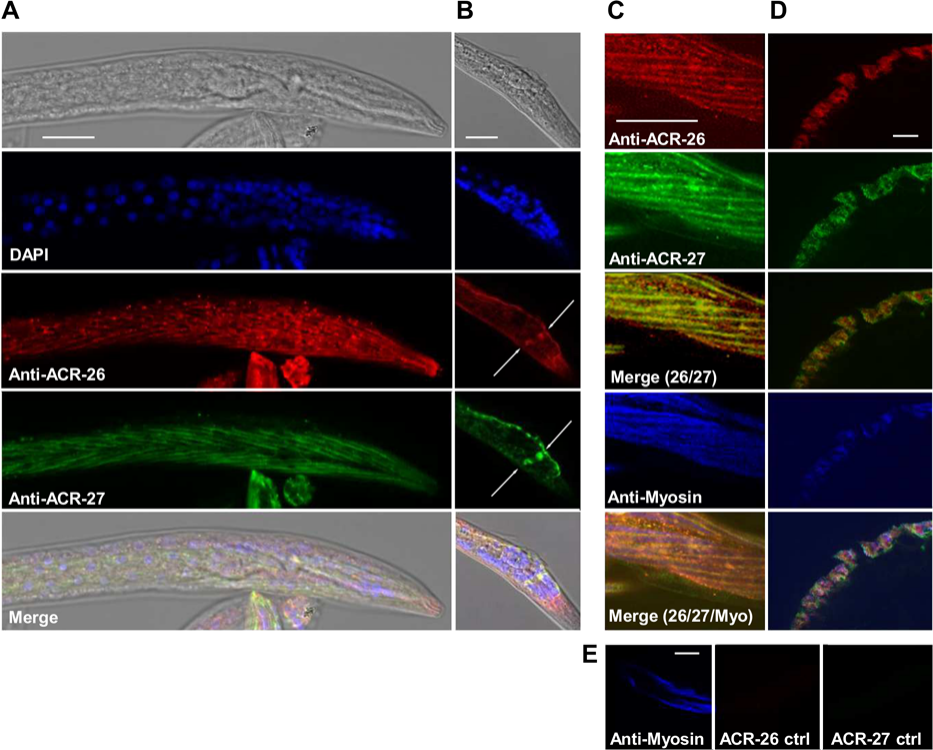
Immunolocalization of Hco-ACR-26 and Hco-ACR-27 in *H*. *contortus*. *H*. *contortus* L2 larval stage and adult males were fixed and incubated with affinity-purified antibodies raised against Hco-ACR-26 and Hco-ACR-27 subunits. Alexa 594- and Alexa 488- labeled secondary antibodies were used to determine the localization of Hco-ACR-26 (red) and Hco-ACR-27 (green) respectively. **(A and B)** Transmitted light and corresponding fluorescent apotome imaging performed on *H*. *contortus* L2 stage. Nematode’s nuclei were stained in blue using DAPI. **Panel A** shows a staining for Hco-ACR-26 and Hco-ACR-27 in striated cells of the nematode. **Panel B** shows that in a deeper focal section, Hco-ACR-26 and Hco-ACR-27 were also found to be expressed in another tissue potentially corresponding to the nerve ring (white arrows). **(C, D and E)** Confocal microscopy performed on L2 stage **(C and E)** and cross section of adult males **(D)**. Body wall muscle cells were stained using primary antibodies raised against the myosin protein and further revealed with secondary Alexa 350-labeled antibodies (blue). **(E)** Negative control performed with anti-Hco-ACR-26 and anti-Hco-ACR-27 pre-adsorbed with their respective peptide antigen. For A, B, C and E anterior region is to the right. The scale bars correspond to 20 μm.

The tissue expression of Hco-ACR-26 and Hco-ACR-27 was then investigated using anti-Hco-ACR-26 and anti-Hco-ACR-27 antibodies (Fig 5). In *H*. *contortus* L2 larvae as well as in cross sections of adult males, an overlapping expression pattern of Hco-ACR-26 and Hco-ACR-27 in body wall muscle cells stained with the anti-myosin antibodies was observed. Noticeably, depending on the focal section, in addition to the “V” shape expression pattern potentially corresponding to the body wall muscle quadrants, in L2 larval stage Hco-ACR-26 and Hco-ACR-27 staining was also found to overlap at the nerve ring level; however this localization remains to be confirmed. All together, these results show that *Hco-acr-26* and *Hco-acr-27* are developmentally co-expressed in muscle.

### Hco-ACR-26 and Hco-ACR-27 form a functional heteropentameric AChR in *Xenopus laevis oocytes*

**Fig 6:**
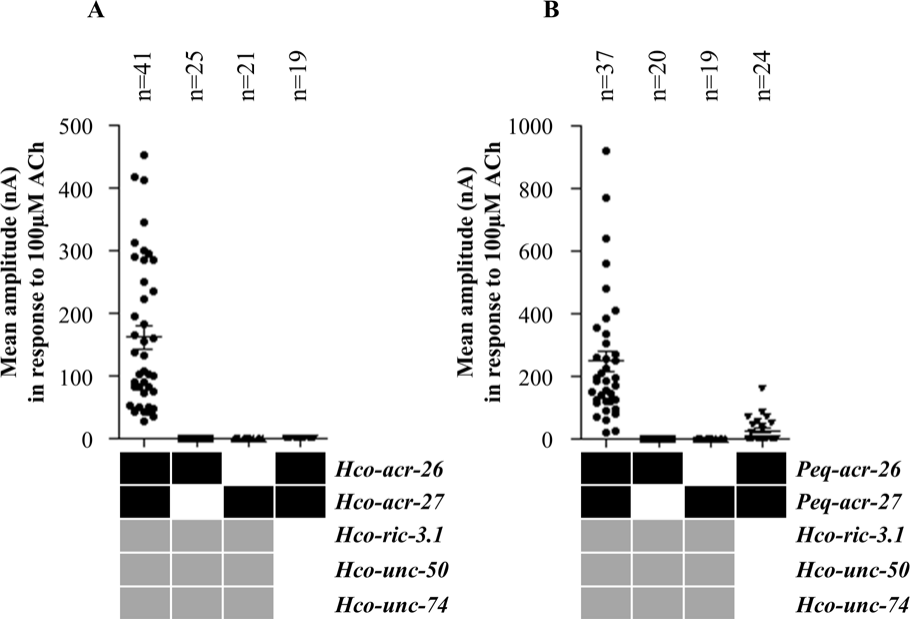
Identification of the minimal subunit combination and ancillary proteins required for the functional expression in *Xenopus* oocyte of AChR containing ACR-26 and ACR-27 from *H*. *contortus* and *P*. *equorum*. For both parasitic nematode species, the robust expression of functional AChRs required the co-injection of cRNAs corresponding to ACR-26 and ACR-27 AChR subunits in combination with the three ancillary factors RIC-3.1; UNC-50 and UNC-74. Currents were measured at the plateau. **(A)** Average plateau values for co-injection of all cRNAs were 161.4±18.67 nA (n = 41) for *H*. *contortus* receptor. **(B)** Average plateau values for co-injection of all cRNAs were 246.8±33.39 nA (n = 37) for *P*. *equorum* receptor. Results are shown in mean ± SEM.

In order to investigate if the overlapping expression contributed to functional receptors, we co-expressed Hco-ACR-26 and Hco-ACR-27 in *Xenopus laevis* oocytes. We have previously reported that the functional expression of nematode muscle AChRs in *Xenopus* oocytes requires the addition of ancillary proteins [24, 25, 30]. Therefore, cRNAs encoding the subunits Hco-ACR-26 and Hco-ACR-27, in combination with three ancillary proteins (*Hco-ric-3*.*1*, *Hco-unc-74* and *Hco-unc-50*) were co-injected into *Xenopus* oocytes. Four days after injection, this combination of cRNAs led to the robust expression of functional receptors responding to 100 μM acetylcholine (ACh) which elicited inward currents in the hundred nA range (Fig 6A). Subsequently, different cRNA combinations were injected into *Xenopus* oocytes in order to determine the minimal subunit combination leading to the expression of a functional receptor and verify the ancillary factor requirement. No current was observed when either Hco-ACR-26 or Hco-ACR-27 alone was expressed in combination with ancillary proteins, suggesting that neither the predicted α-Hco-ACR-26 nor non-α-Hco-ACR-27 is able to form a functional homomeric AChR. Moreover, a combination containing both Hco-ACR-26 and Hco-ACR-27 subunits in the absence of ancillary proteins failed to give rise to a functional AChR. Even though their individual role remains to be further investigated; this result highlights an important requirement for ancillary proteins that are essential for the functional expression of other classes of AChRs in *Xenopus* oocytes. We conclude that Hco-ACR-26 and Hco-ACR-27 are able to combine together to form functional heteropentameric receptor when co-expressed with ancillary proteins in the *Xenopus* oocyte. This novel receptor was named Hco-26/27.

### Functional expression of Peq-26/27 AChR in *Xenopus* oocyte

In order to determine if ACR-26 and ACR-27 subunits from other parasitic nematodes species could also form functional receptors when expressed in *Xenopus* oocyte, *Peq-acr-26* and *Peq-acr-27* cRNAs from the distantly related *P*. *equorum* (Clade III) were co-injected in combination with *Hco-ric-3*.*1*, *Hco-unc-74* and *Hco-unc-50* cRNAs. Four days after injection, we observed robust expression of a functional AChR responding to 100 μM ACh (Fig 6B). As observed for Hco-26/27, neither the expression of Peq-ACR-26 nor Peq-ACR-27 alone resulted in the expression of a functional AChR. However, in contrast with Hco-26/27 the co-expression of ancillary proteins was found not to be absolutely required for functional Peq-26/27 but in their absence its expression was drastically reduced. This novel heteropentameric AChR was named Peq-26/27.

### Pharmacological characterization of Hco-26/27 and Peq-26/27 receptors

**Fig 7:**
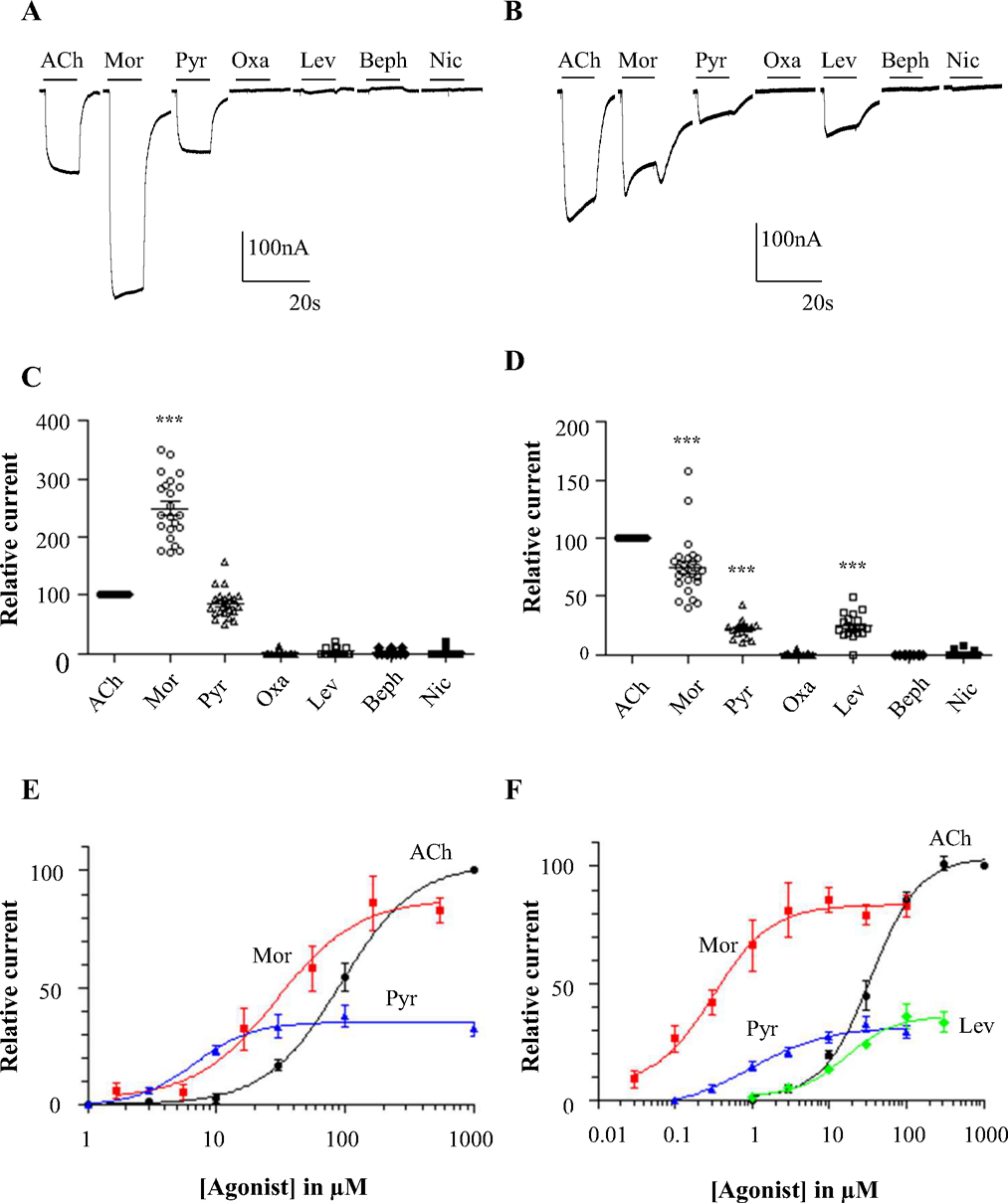
Pharmacological profiles of Hco-26/27 and Peq-26/27. (A and B) Representative recording traces from a single oocyte expressing Hco-26/27 (A) or Peq-26/27 (B) challenged with 100 μM ACh and 100 μM of different anthelmintic compounds (morantel (Mor), pyrantel (Pyr), oxantel (Oxa), levamisole (Lev), bephenium (Beph) and nicotine (Nic)). The bars indicate the time period of the agonist application. (C and D) Scatter plot (mean ± SEM) of normalized currents elicited by 100 μM of anthelmintic compounds on Hco-26/27 (C) or Peq-26/27 (D). Currents have been normalized to and compared with 100 μM ACh currents. Paired Student’s t-test, ***p<0.001. (E) Dose-response relationships of Hco-26/27 for the agonists ACh (black circle, n = 6), Mor (red squares, n = 8) and Pyr (blue triangles, n = 6). Responses are all normalized to the response to 1 mM acetylcholine. EC_50_ are 80.1±1.1 μM, 29.0±1.3 μM and 6.8±1.3 μM for ACh, Mor and Pyr respectively. (F) Dose-response relationships of Peq-26/27 for the agonists ACh (black circle, n = 7), Mor (red squares, n = 9), Pyr (blue triangles, n = 10) and Lev (green lozenges, n = 6). Responses are all normalized to the response to 1 mM acetylcholine. EC_50_ are 34.9±1.1 μM; 0.32±0.26 μM; 0.98±0.26 μM and 16.7±1.3 μM for ACh, Mor, Pyr and Lev respectively.

The pharmacological profiles of Hco-26/27 and Peq-26/27 receptors were established using ACh in comparison with a set of cholinergic agonists used as anthelmintic compounds including morantel (Mor), pyrantel (Pyr), oxantel (Oxa), levamisole (Lev), bephenium (Beph) and nicotine (Nic), (Fig 7). The Hco-26/27 receptor was highly responsive to 100 μM Mor (249.2±11.5% of ACh response) or 100 μM Pyr (81.01±6.21% of ACh response) with a fast activating response that quickly reached a plateau (Fig 7A and 7C) but insensitive to the other cholinergic agonists Oxa, Lev, Beph and Nic. The subunit stoichiometry has been previously reported to impact the pharmacological properties of some nematode AChRs expressed in *Xenopus* oocytes [27]. Providing fivefold more Hco-ACR-26 did not produce a significantly different response following application of 100 μM ACh, Pyr, Mor, Oxa, Nic, Lev, or Beph whereas a fivefold increase of Hco-ACR-27 led to a reduction of Mor response (2 fold Imax reduction p<0.001) that remained higher than for Pyr or ACh in comparison with equal subunit ratios (S2 Table).

Strikingly, Peq-26/27 receptor was also found to be responsive to Mor (74.5±4.9% of ACh response), providing the first evidence that despite their wide phylogenetic distance, the sensitivity to Mor represents a conserved feature of the 26/27 subunit containing AChR from Clade V and Clade III nematodes (Fig 7B and 7D). Moreover, Peq-26/27 was responsive to Pyr (21.8±2.1% of ACh response) and insensitive to Nic as for Hco-26/27 whereas it was sensitive to Lev (24.5±2.4% of ACh response).

Hco-26/27 and Peq-26/27 dose responses for ACh, Mor and Pyr and additionally Lev for Peq-26/27 are shown in Fig 7E and 7F. All responses were normalized to the maximal response to 1 mM ACh. Sensitivities of Hco-26/27, for Mor (EC_50_ 29.0±1.3 μM; n = 8) and Pyr (EC_50_ 6.8±1.3 μM; n = 6) were higher than for the natural ligand ACh (EC_50_ 80.1±1.1 μM; n = 6). The same was true for Peq-26/27: Mor (EC_50_ 0.98±0.26 μM; n = 9), Pyr (EC_50_ 0.32±0.26 μM; n = 10) and ACh (EC_50_ 34.9±1.1 μM; n = 7). The Peq26/27 sensitivity for lev, (EC_50_ 16.7±1.3 μM (n = 6)) was much lower than either Mor and Pyr.

In *C*. *elegans*, functional null mutants for genes encoding each of the essential subunits of the L-AChR (*unc-38*, *unc-63* or *unc-29*) are resistant to high concentrations of Mor suggesting that this drug could target the L-AChR [38]. Therefore, we also investigated the potential Mor effect on the two recombinant L-AChR subtypes identified from *H*. *contortus*: Hco-L-AChR-1 (Hco-UNC-38, Hco-UNC-63, Hco-UNC-29.1, Hco-ACR-8) and Hco-L-AChR-2 (Hco-UNC-38, Hco-UNC-63, Hco-UNC-29.1) [25] (S3 Fig). In contrast with Lev, to which Hco-L-AChR-1 is very responsive, we noted that a high dose of 100 μM Mor only induced a weak response within the same range as observed for Nic or Pyr application. In a similar way, Hco-L-AChR-2 that is highly responsive to Pyr was also less activated by 100 μM Mor. Interestingly, in comparison with Hco-26/27, Hco-L-AChR-1 and Hco-L-AChR-2 harbor a higher sensitivity for Mor (EC_50_ 0.41±0.23 μM and EC_50_ 3.2±1.2 μM respectively). Note that Mor concentrations above 30 μM had a potential channel-block effect on Hco-L-AChR-1. Importantly, for both Hco-L-AChR-1 and Hco-L-AChR-2 the maximal normalized responses elicited by Mor, remain much lower than for Hco-26/27. These observations suggest that distinct body wall muscle AChR subtypes might underlie the potent efficacy of Mor on *H*. *contortus* and support the hypothesis that Hco-26/27 represents a preferred target for Mor.

### The co-expression of ACR-26 and ACR-27 subunits from parasitic nematodes in body wall muscle of *C*. *elegans* increases its sensitivity to morantel and pyrantel

In the present study we used the nematode *C*. *elegans* as a heterologous expression system with two main objectives: first to get some new insights into the functional expression of Hco-26/27 and Peq-26/27 *in-vivo* and second to evaluate the relevancy of using *C*. *elegans* expressing AChR of parasitic nematodes as a potential drug screening tool.

Taking advantage of the absence of *acr-26* and *acr-27* homologs in the *C*. *elegans* genome, we used wild type N2 worms to express these two parasitic nematode AChR subunits. Because ACR-26 and ACR-27 subunits from *H*. *contortus* were found to be co-localized in muscle cells, we expressed these two subunits under the control of the *C*. *elegans myo3* body wall muscle promoter. For Peq-ACR-26 and Peq-ACR-27, even though the localization of these subunits in *Parascaris equorum* remains to be investigated in more detail, we also used *myo3* as a promoter in order to compare the functional impact of Hco-26/27 and Peq-26/27 on both motility and drug sensitivity in transgenic *C*. *elegans*.

In order to set up an experimental design aimed at detecting modulation of drug sensitivity in transgenic *C*. *elegans* expressing either Hco-26/27 or Peq-26/27, we first performed dose-response thrashing assays on *C*. *elegans* N2 using Mor, Pyr and Lev. We used drug-induced reduction in the periodic thrashing to define IC_50_ (S4A Fig) and subsequently compared the threshold doses that caused significant reduction of motility relative to untreated worms (S4B Fig). *C*. *elegans* N2 thrashes were recorded after a 10 minute exposure to Mor, Pyr and Lev, corresponding to the mean observed time for which the three drugs have a strong effect on worm locomotion. *C*. *elegans* N2 were found to be more sensitive to Lev (IC_50_ = 7.0±1.1 μM) than either Mor (IC_50_ = 44.1±1.0 μM) or Pyr (IC_50_ = 107.1±1.2 μM) as previously observed [38]. For Lev, Mor and Pyr, we determined that 5 μM, 40 μM and 50 μM corresponded respectively to the minimal drug concentrations required to cause a significant reduction in wild-type motility.

**Fig 8:**
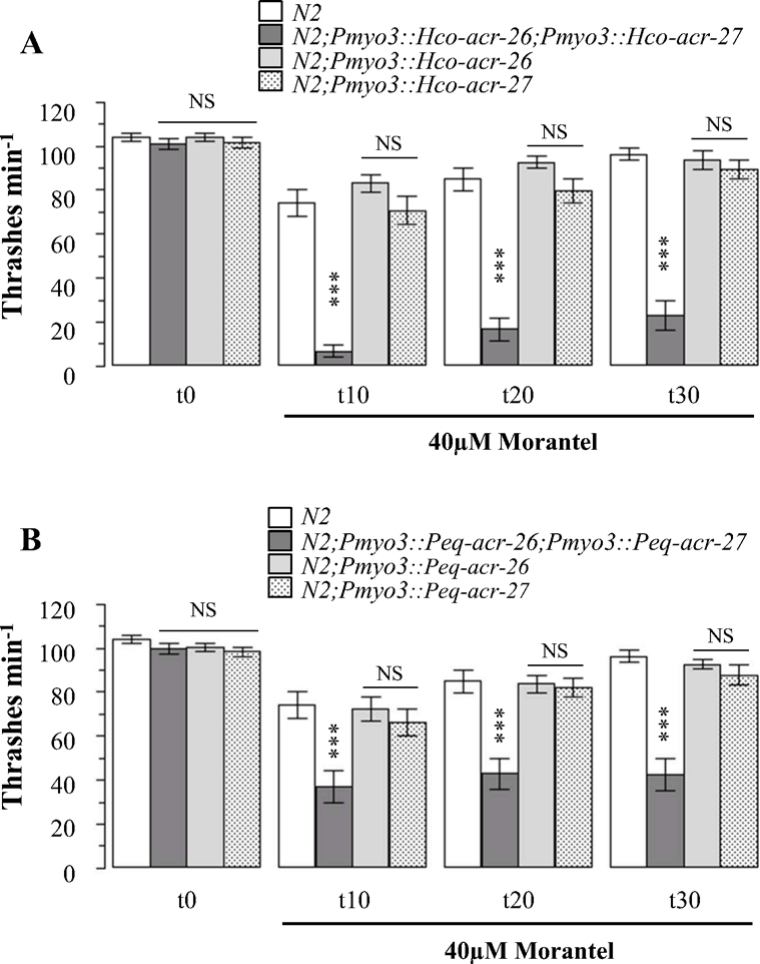
Co-expression of ACR-26 and ACR-27 in *C*. *elegans* increases its morantel sensitivity. Thrashing assays were performed during 30 minutes with t0 corresponding to basal movements. For each *C*. *elegans* expressing parasitic nematode ACR-26 or ACR-27 alone or in combination, two independent lines were used with >12 worms per lines. Thrashing assays performed with 40 μM morantel on N2 (WT) and transformed worms expressing ACR-26 and ACR-27 subunits (alone or in combination) from *H*. *contortus* (A) or *P*. *equorum* (B). All results are expressed as mean ± SEM. Using *C*. *elegans* N2 as reference, statistical analysis was performed using the One-Way ANOVA with Tukey’s Multiple Comparison Test with ***p<0.001, NS, not significant.

Transgenic *C*. *elegans* expressing Hco-26/27, Peq-26/27 or single subunits alone were not significantly affected in their locomotion compared to N2 (Fig 8). Strikingly, worms expressing Hco-26/27 or Peq-26/27 showed a strong reduction of their thrashing motility when exposed to 40 μM Mor (93.3% and 62.7% after a 10 minutes exposure) whereas *C*. *elegans* expressing individual subunits alone were indistinguishable from wild-type (N2) worms (Fig 8). All together, these results provide the first evidence that co-expression of ACR-26 and ACR-27 subunits from the parasitic nematode species *H*. *contortus* and *P*. *equorum* is required to obtain a functional AChR sensitive to Mor in *C*. *elegans* body wall muscle. In addition, these observations also suggest that single ACR-26 or ACR-27 subunit from the parasitic species do not assemble into functional Mor-sensitive AChRs as either homopentamers nor as heteropentamers with other *C*. *elegans* subunits present in body muscle cells.

Importantly, in *C*. *elegans* expressing Hco-26/27 or Peq-26/27, 50 μM Pyr also induced a reduction of motility in comparison with N2 worms (S5 Fig), with percentage reduction of thrashes min^-1^ ranging from 65.4% for Hco-26/27 to 61.6% for Peq-26/27 after 10 minutes of drug exposure. These results further validate the pharmacological properties of Hco-26/27 or Peq-26/27 determined in *Xenopus* oocytes. It is noteworthy that for all *C*. *elegans* strains tested here, the maximal effects of Mor and Pyr were observed after 10 minutes of drug exposure and that worms began to recover after longer exposure times (Fig 8 and S5 Fig). However, for both drugs, *C*. *elegans* expressing Hco-26/27 or Peq-26/27 AChRs still had a strong reduction of motility in comparison with wild type worms whatever the exposure time.

In order to investigate whether the expression of Hco-26/27 or Peq-26/27 receptors could impact the sensitivity of *C*. *elegans* to other drugs targeting its endogenous muscle AChR, thrashing assays with Lev (5 μM) were performed on the transgenic *C*. *elegans* (S5 Fig). Expression of transgenic Hco-26/27 or Peq-26/27 produced no significant change in sensitivity to 5 μM Lev (S5 Fig) suggesting that endogenous L-AChRs remained responsible for sensitivity to Lev, despite the fact that the Peq-26/27 was sensitive to Lev in *Xenopus* oocytes. Note that the native *C*. *elegans* L-AChR is more sensitive to Lev (EC_50_ 10 μM) [24] than the Peq-26/27 (EC_50_ 17 μM) and so we could speculate that the lack of any difference in sensitivity to Lev in *C*. *elegans* expressing Peq-26/27 is because the native L-AChR may compete for Lev binding.

## Discussion

### Diversity of nematode muscle acetylcholine receptors and subunit genes

In the present study we report that ACR-27, a non-α subunit closely related to ACR-26, is able to combine with ACR-26 to form a novel muscle receptor subtype in parasitic nematodes. Nematode muscle AChRs are prime pharmacological targets for anthelmintic drugs. They have been extensively studied in the model nematode *Caenorhabditis elegans* providing a strong basis to investigate their counterparts in parasitic nematode species. Single channel recording experiments revealed at least two main muscle AChR subtypes in *C*. *elegans*: the Lev-sensitive AChR (L-AChR) and the Nic-sensitive AChR (N-AChR) [39]. The *C*. *elegans* L-AChR is a heteropentameric channel made of 3 α-subunits (UNC-38; UNC-63, LEV-8) and two non-α-subunits (LEV-1; UNC-29) [24, 40, 41, 42, 43]. This receptor is sensitive to the cholinergic agonists Lev, Mor and Pyr and is insensitive to nicotine [24, 44]. The *C*. *elegans* N-AChR is a homopentameric channel containing ACR-16 α-subunits that is sensitive to nicotine and insensitive to Lev [22, 39, 45]. In parasitic nematode species, single channel recordings have revealed a greater diversity of muscle AChR subtypes. In the pig parasitic nematodes *Oesophagostomum dentatum* (Clade V) and *Ascaris suum* (Clade III), Lev was found to activate up to three populations of channels separated by their conductance and designated as G25 (small conductance: 22 pS), G35 (intermediate conductance: 33 pS) and G45 (large conductance: 45 pS), [46, 47]. In addition to these three channel populations, a fourth Lev-activated AChR subtype with a conductance of 40 pS was also reported in *O*. *dentatum* (Clade V), [46]. The molecular basis of L-AChR diversity in parasitic nematodes has been investigated using the *Xenopus* oocyte expression system [48]. For *A*. *suum*, only two AChR subunits homologous to UNC-29 and UNC-38 were sufficient to reconstitute functional L-AChRs [27]. In *H*. *contortus* and *O*. *dentatum* we previously reported that different combinations of AChR subunits homologous to UNC-38 UNC-63, UNC-29 and ACR-8 led to the functional expression of L-AChR subtypes with different pharmacological properties [25, 30].

Recently, Bennett *et al*. reported the identification of a novel α-subunit encoded by the *acr-26* gene [28]. This gene was found to be present in parasitic nematode species from Clade III, IV and V and was absent form free-living and plant parasitic nematodes. In *A*. *suum*, immunolocalization experiments revealed a muscular expression of ACR-26. In the present work, we demonstrate that in addition to ACR-26, parasitic nematode species possess an additional AChR subunit encoded by the *acr-27* gene providing new insights into our knowledge of AChR diversity and specificity from parasitic nematodes.

### *ACR-27* is a nematode-specific AChR subunit

Our analysis of genomic data from 88 nematode species revealed that *acr-27* homologs are present in parasitic species from Clade III, IV and V that also possess an *acr-26* gene and are essentially absent in free-living and plant parasitic nematodes from the same clades. Whereas *acr-26* homologous genes could be identified in other invertebrate genomes such as the molluscs *Aplysia californica*, *Crassostrea gigas*, *Lottia gigantean* and the arthropod *Ixodes scapularis*, we found no evidence for *acr-27* homologs outside the phylum Nematoda. Even though we cannot rule out that homologs could exist in other animal phyla, our results suggest that *acr-27* is nematode-specific. Because *acr-26* and *acr-27* are close homologues that cluster together but separately from the other AChR subunits described in *C*. *elegans and H*. *contortus* [32], it is tempting to hypothesize that *acr-27* genes could have arisen from an ancestral *acr-26* gene. However, if the origin of *acr-27* from *acr-26* occurred prior to the separation of molluscs from the ecdysozoa, it is therefore difficult to explain why this receptor class is now found almost exclusively in parasitic nematodes and apparently nowhere else. Importantly, parasitism is thought to have arisen from multiple independent events in nematodes [49]. Therefore, we might speculate that the conservation of *acr-26* and *acr-27* genes in parasitic species from distinct clades and their loss in free-living or plant parasitic species from the same clade could reflect a potential involvement of the ACR-26/ACR-27 receptor in animal parasitism. Noticeably, we found two major but non-exclusive exceptions to this postulate. We identified homologs of *acr-26* and *acr-27* genes in the Clade IV free-living species *Panagrellus redivivus* and *Rhabditophanes sp* KR3021. However it is noteworthy that *Rhabditophanes sp* KR3021 is an example of a reversal from a parasitic to a non-parasitic life style [50].

### A novel class of heteropentameric AChR in parasitic nematodes

In the present study, we raised the hypothesis that ACR-26 and its closest homolog ACR-27 could form functional channel in nematodes. This hypothesis was supported by the evidence that two closely related AChR subunits from the DEG-3 group (i.e. DEG-3 and DES-2 from *C*. *elegans* and *H*. *contortus*) are able to form functional channels when co-expressed in *Xenopus* oocytes [23, 51].

Both *Hco-acr-26* and *Hco-acr-27* are co-expressed in both free-living and parasitic stages, indicating that they play a role in multiple developmental stages. Their respective transcripts and proteins co-expressed in body wall muscles and nerve ring of *H*. *contortus* suggesting they may combine in the same AChR at the neuromuscular junction. Clearly, further investigations using antibodies raised against synaptic proteins of *H*. *contortus* would be required to address this question.

In the present work we were able to reconstitute a new class of functional AChR in *Xenopus* oocytes by co-expressing ACR-26 and ACR-27 from either *H*. *contortus* or the distantly related *P*. *equorum* and a set of three ancillary proteins that have been shown to be required previously for the functional expression of recombinant L-AChRs from *C*. *elegans*, *H*. *contortus* and *O*. *dentatum* [24, 25, 30]. These ancillary factors are: RIC-3, a chaperone protein involved in the subunit assembly or maturation of L- and N-AChRs in *C*. *elegans* [52–54]; UNC-74 a thioredoxin-related protein required for the proper folding of L-AChR subunits and closely related to the human TMX3 protein [55] and UNC-50 a transmembrane protein involved in the regulation of L-AChR trafficking [56]. Ancillary factors were an absolute requirement for the functional expression of the Hco-26/27 receptor, unlike Peq-26/27 where a decreased expression of the receptor could be achieved without the addition of ancillary factors. This is similar to a study by Williamson *et al*. which showed that the L-AChR from *A*. *suum* could be expressed in *Xenopus* oocytes without ancillary proteins [27]. Therefore, the putative role of the ancillary proteins for the assembly, processing and membrane targeting of ACR-26/27-containing receptors *in vivo* in different species of nematode may vary and remains to be resolved.

For both *H*. *contortus* and *P*. *equorum*, we demonstrated that the expression of either the ACR-26 or ACR-27 subunit alone with the three ancillary proteins in oocytes never led to the expression of functional AChR. For Hco-ACR-26, this is in accordance with the Bennett *et al*.’s observation, but it is noteworthy that in their study the authors only used the *Xenopus* RIC-3 as an ancillary protein [28]. However, in contrast to Bennett *et al*. who reported the occasional expression of a homomeric channel for the *Ascaris suum* ACR-26 subunit, in our hands, the closely related *P*. *equorum* ACR-26 subunit alone never gave a functional homomeric AChR. Therefore our results suggest that Hco-ACR-26 and Peq-ACR-26 preferentially combine with an additional subunit such as Hco-ACR-27 or Peq-ACR-27 respectively to form functional heteropentameric AChRs. Similarly, the expression of either Hco-ACR-27 or Peq-ACR-27 alone with the three ancillary proteins did not lead to expression of a functional receptor. This is consistent with suggestions that non-α subunits are not able to form homopentameric channels.

The pharmacological characterization of Hco-26/27 revealed some striking features. Though this receptor was found to be relatively insensitive to the prototypical nematode muscle AChR agonists Lev and Nic, it was found to be highly sensitive to the tetrahydropyrimidines Mor and Pyr. Noticeably, Mor and Pyr had a better potency than the endogenous agonist ACh. In a previous study, we reported that the recombinant *H*. *contortus* levamisole-sensitive receptor Hco-L-AChR-2 was also highly sensitive to Pyr [25]. Here we demonstrate that in sharp contrast with Hco-26/27, Hco-L-AChR-2 was poorly responsive to Mor in comparison to Pyr. Even though we cannot rule out that *H*. *contortus* expresses other muscle AChR subtypes that might be highly responsive to Mor this result suggests that Hco-26/27 represents a major target for Mor. Ideally, single channel experiments in muscle of *H*. *contortus* could be helpful to determine the conductance induced by Mor and Pyr to investigate the potential diversity of their receptors *in vivo*. Interestingly, Peq-26/27 and Hco-26/27 were found to share the same major pharmacological properties with the exception of Lev for which Peq-26/27 was found to be sensitive. However, it is noteworthy that Lev has a much lower potency on Peq-26/27 than Mor and Pyr. Interestingly, Peq-26/27 EC_50_ values for ACh, Pyr and Mor were found to be smaller than their *H*. *contortus* counterparts indicating a better potency of these ligands on Peq-26/27 than for Hco-26/27. All together our results suggest that AChR containing ACR-26 and ACR-27 subunits represent a novel nematode AChR subtype that could be a molecular target for Mor and Pyr.

### *C*. *elegans* as a functional tool to express parasite drug targets (AChRs)

Even though genetic transformation has been reported in some parasitic nematodes such as *Parastrongyloides*, *Strongyloides spp* [57–59] and *Brugia malayi* [60, 61], genetic engineering remains an elusive goal for a large majority of nematode parasites. In plant parasitic nematodes such as *Globodera pallida*, *Heterodera glycines* and *Meloidogyne spp*, RNAi has been shown to be efficient and reliable and it currently represents a promising approach for the development of a novel control method against these phytopathogens [62]. However, in animal parasitic nematodes, RNAi is found to be much more challenging because of its inconsistent efficacy [63]. In this respect, the use of *C*. *elegans* as a heterologous expression system for parasitic nematode genes represents an alternative for their functional analysis [64]. This model has been successfully exploited to validate anthelmintic targets from parasitic nematodes as well as drug resistance mechanisms. *C*. *elegans*, resistant to benzimidazoles through a mutation disrupting the *ben-1* gene are rescued when transfected with the isotype-1 *β-tubulin* gene from *H*. *contortus* [65]. Normal locomotion can be restored to *C*. *elegans* mutants lacking a functional *avr-14* GluCl subunit by expressing its ortholog from *H*. *contortus* (*Hco-avr-14a* and *Hco-avr-14b*) [66]. Expression of *H*. *contortus avr-14b* and *glc-6* was able to completely restore ivermectin sensitivity in *C*. *elegans* triple mutant (DA1316) lacking functional *avr-14*, *avr-15* and *glc-1* genes [67]. Noticeably, *Hco-glc-6* has no ortholog in *C*. *elegans* and this result highlights the value of this expression system to decipher anthelmintic targets that are specific to parasitic nematode species. Recently, expression of the ortholog of *slo-1* from the parasitic nematodes *Ancylostoma caninum* or *Cooperia onchophora* into a *C*. *elegans slo-1* loss-of-function mutant restored sensitivity to emodepside [68–69].

Here we report for the first time the expression in *C*. *elegans* of a novel class of AChR for which no ortholog could be identified in its genome. Using Mor and Pyr sensitivity to define this new receptor class, we confirmed the functional expression of 26/27 receptors from phylogenetically distant parasitic nematode species (i.e. *H*. *contortus*, Clade V and *P*. *equorum*, Clade III) in *C*. *elegans* wild-type genetic background. Despite the fact that *acr-26* and *acr-27* homologs are absent from the *C*. *elegans* genome, the successful functional expression of Hco-26/27 and Peq-26/27 provides strong evidence that the molecular machinery involved in AChR subunit assembly and trafficking is conserved between nematode species.

In *C*. *elegans*, single channel recordings performed on muscle cells showed that both Mor and Pyr target the L-AChR [44]. In accordance, *C*. *elegans* mutants for *unc-38*, *unc-63* or *unc-29* L-AChR subunit genes were found to be resistant to a high concentration (1 mM) of Mor, Pyr and Lev [38]. In the present work, we showed that transgenic *C*. *elegans* expressing Hco-26/27 or Peq-26/27 have a drastically increased sensitivity to Mor and Pyr providing additional evidence that ACR-26/ACR-27 receptors represent pharmacological targets for these drugs in both parasitic nematode species. Therefore, based on their validated pharmacological properties we propose to name this novel class of morantel-sensitive AChR, the M-AChR, to distinguish them from the L-AChR and N-AChR respectively.

In summary, the results obtained in the present study confirm the ability of ACR-26 and ACR-27 subunits from phylogenetically distant species to form a functional M-AChR in *C*. *elegans* and also highlight the value of *C*. *elegans* expressing AChR from parasitic nematodes as a relevant screening tool for the discovery of novel anthelmintics. Indeed, *C*. *elegans* expressing ACR-26/ACR-27 were easily amenable to efficient motility assays.

### New insights into the Morantel mode of action

Even though we cannot exclude that other muscle AChRs from parasitic nematodes might constitute a preferential pharmacological target for Mor *in-vivo*, our results provide new insights into the mode of action of this anthelmintic. Mor has been used to control gastro-intestinal parasitic nematodes from ruminants including Clade V parasitic nematode species such as *H*. *contortus*, *Teladorsagia circumcincta*, *Trichostrongylus colubriformis* and *Ostertagia ostertagi* [70]. Interestingly, Mor was also found to be effective *in vivo* or *in vitro* against the Clade III parasitic species *P*. *equorum* and the human parasitic species *Brugia malayi* respectively [71, 72]. It is noteworthy that in addition to *H*. *contortus* and *P*. *equorum*, for most of these parasitic nematode species we could find evidence in genomic databases for the presence of *acr-26* and *acr-27* homologous genes. Because Lev and Mor side resistance has been observed in field or laboratory isolates in the trichostrongylid species *H*. *contortus*, *T*. *colubriformis*, *T*. *circumcincta* and *O*. *ostertagi*, it was commonly accepted that resistance to these cholinergic agonists was co-inherited [73–79]. Controversially, other studies reported that in field or laboratory-selected isolates of *T*. *colubriformis*, Mor resistant worms remained sensitive to Lev [80–81], suggesting potential distinct pharmacological targets preferentially activated by these drugs. Therefore, our results are in accordance with this latter suggestion. Undoubtedly, further investigations of potential polymorphisms in *acr-26* and/or *acr-27* genes from parasite isolates presenting single resistance to Mor would be of particular interest to explore this hypothesis.

In conclusion, we provide here: 1) the first evidence that ACR-26/ACR-27 AChR subunits are components of the M-AChR, a novel class of AChR in nematodes, paving the way for their detailed characterization in a wide range of parasitic species; 2) a proof of concept that M-AChRs can be heterologously expressed in different systems, laying the basis for target-based drug screening; 3) new insight about the mode of action of morantel, underscoring its interest for potential drug combination strategies.

## Methods

### Ethics statement

All animal care and experimental procedures were conducted in strict accordance with the European guidelines for the care and use of laboratory animals and were approved by the ethical committee from Indre et Loire under experimental agreement 6623 provided by the French Veterinary Services.

### Nematodes

*Haemonchus contortus* experiments were performed on the inbred-susceptible-Edinburgh (ISE) isolate [82] as previously described [83]. Adult *Parascaris equorum* worms were obtained from naturally infected horses from the INRA Tours research center. The presence of *P*. *equorum* in horses was monitored by coproscopy and living adult worms were collected in host feces 6 hours after an ivermectin treatment. Parasitic nematode samples were stored in RNA later solution (Ambion) at -80°C until used. *Caenorhabditis elegans* experiments were carried out on the Bristol N2 wild-type strain obtained from the *Caenorhabditis* Genetics Center (CGC).

### cDNA synthesis

For free-living stages of *H*. *contortus*, total RNA was prepared from 50μL of pelleted eggs, L2, L3 or XL3 stages. For parasitic stages of *H*. *contortus* total RNA was prepared from 250 L4 or 10 adult worms (males or females) respectively. For *Parascaris equorum*, a cross-section (5 mm thick) from the mid body region of an individual adult worm was collected for subsequent RNA preparation. For *C*. *elegans*, total RNA was prepared from 12 adult worms.

Frozen samples were ground in liquid nitrogen and homogenized in Trizol reagent (Invitrogen, Carlsbad, CA, USA) and total RNA was isolated according to the manufacturer’s recommendations. RNA pellets were dissolved in 25 μL of RNA secure resuspension solution (Ambion, Austin, TX, USA) and DNase-treated using the TURBO DNA-free kit (Ambion). RNA concentrations were measured using a nanodrop spectrophotometer (Thermo Scientific, Waltham, MA, USA). First-strand cDNA synthesis was performed on 1μg of total RNA using the superscript III reverse transcriptase (Invitrogen, Carlsbad, CA, USA) according to the manufacturer’s recommendations.

### Cloning of complete coding cDNA sequences of *acr-26* and *acr-27* from *H*. *contortus* and *P*. *equorum*

Complete coding sequences corresponding to *Hco-acr-26*, *Hco-acr-27*, *Peq-acr-26* and *Peq-acr-27* genes were identified using first-strand cDNA prepared from adult worms as template. PCR amplifications were performed with the proofreading Phusion High fidelity Polymerase (New England Biolabs) following the manufacturer’s recommendation. Primer sequences are reported in S3 Table. Amplicons were cloned into the pCR4Blunt-TOPO vector (Lifestechnologies) and sequenced by GATC biotech (Konstanz, Germany).

The amplification of *Hco-acr-27*, *Peq-acr-26* and *Peq-acr-27* cDNA 5’ ends was obtained by semi-nested PCRs using the splice leader primer SL1 (GGTTTAATTACCCAAGTTTGAG) in combination with internal reverse primers. For each sequence, the corresponding 3′cDNA end was identified by 3′RACE PCR using the GeneRacer kit (Invitrogen) with two rounds of PCR. Primer sequences were designed based on predicted coding sequence available in genomic databases: *Hco-acr-27*: supercontig 58123 (http://www.sanger.ac.uk/cgi-in/blast/submitblast/h_contortus) assembled supercontigs database (21/08/08); *Peq-acr-26*: PEQ scaffold 0005908 (http://parasite.wormbase.org/Parascaris_equorum_prjeb514); *Peq-acr-27*: PEQ scaffold 0021885 (http://parasite.wormbase.org/Parascaris_equorum_prjeb514). Subsequently, complete coding sequence from *Hco-acr-26*, *Hco-acr-27*, *Peq-acr-26* and *Peq-acr-27* were amplified with a forward primer including the first ATG codon in combination with a reverse primer including the first stop codon. For *Hco-acr-26* primers were designed on the sequence available in GenBank under accession number EU006791 whereas for *Hco-acr-27*, *Peq-acr-26* and *Peq-acr-27* primers were designed on sequences obtained after cDNA 5’ and 3’ end identification. *Hco-acr-27*, *Peq-acr-26* and *Peq-acr-27* complete coding sequences were deposited to GenBank under the accession number: KC790461; KP756902 and KP756903 respectively.

### Sequence analysis

Database searches were performed with the tBLASTn or BLASTP algorithms [84]. Nematode genomic databases investigated in the present study are summarized in S1 Table. Protein sequences were aligned using MUSCLE [36]. Signal peptide predictions were carried out using the SignalP 3.0 server [85] and membrane-spanning regions were predicted using the SMART server [86].

Phylogenetic analysis was performed on deduced amino-acid sequence predicted from genomic sequences available in databases (see S1 Table) or cloned cDNA sequences (i.e. Hco-ACR-26; Hco-ACR-27, Peq-ACR-26; Peq-ACR-27 and Asu-ACR-26). Sequence from the signal peptide, the intracellular loop and C-terminal tail that could not be aligned unambiguously were removed. Maximal likelihood phylogeny reconstruction was performed using PhyML V20120412 (https://github.com/stephaneguindon/phyml-downloads/releases) and significance of internal tree branches was estimated using bootstrap resampling of the dataset 100 times.

### Expression of *Hco-acr-26* and *Hco-acr-27* through the *H*. *contortus* lifecycle

PCR was carried out on *H*. *contortus* first strand cDNA prepared from eggs, L2, L3, XL3, L4 (5dpi), adult males and adult females. PCR reactions were carried out in a final volume of 20 μl, containing 100 ng of first strand cDNA, 1 unit of GoTaq polymerase (Promega), 0.25 mM dNTPs each and 0.3 μM of each primer. The reaction mixture was denatured by heating to 94°C for 5 min, followed by 34 cycles of 94°C for 45 sec, 56°C for 45 sec, 72°C for 45 sec. A final extension step was performed at 72°C during 5 min. *H*. *contortus* GAPDH (HM145749) was used as the reference transcript. Primer sequences are provided in S3 Table. PCR products were subjected to electrophoresis through a 1.5% agarose gel.

In order to analyze the relative expression of *Hco-ACR-26 and Hco-acr-27* in L3 and adult stages of *H*. *contortus*, quantitative RT-PCR experiments were carried out with specific forward and reverse primers designed either in identified sequences. Primers sequences used for QRT-PCR experiments are provided in S3 Table. QRT-PCR experiments were performed by monitoring the increase of fluorescence of IQ SYBR Green (Bio-Rad, Hercules, CA, USA) with the Rotor-Gene 3000 (Corbett Research, Sydney Australia). The relative fold change of gene expression was calculated with the 2∆CT method using the Gene Expression Analysis software (Bio-Rad, Hercules, CA, USA). The *H*. *contortus gapdh*, *actin*, and *β-tubulin* (isotype 1) were used as reference genes. The data were presented as fold changes in mean ± SEM of mRNA expression compared to the mRNA expression levels for *Hco-acr-26* normalized to 1 for each stage.

### *In-situ* hybridization experiments

Specific primers designed from the *Hco-acr-26* and *Hco-acr-27* cDNA sequence were used to amplify 300 and 258 bp products. Primer sequences are presented in S3 Table. These PCR products were used to synthesize digoxigenin-labeled antisense and sense single strand cDNA probes by asymmetric PCR as previously described [87]. *In-situ* hybridizations were performed on XL3 from *H*. *contortus* as follow: XL3 were permeabilized and fixed using procedures adapted from *C*. *elegans* protocols [88]. Briefly, 75 μL of pelleted *H*. *contortus* XL3 were placed between two Superfrost plus slides and frozen on dry ice for 20 min. The slide sandwich was cracked by swiftly pulling apart the two slides and the worms were thawed and incubated for 16 hours at 4°C in a PBS 1X solution with 4% PFA. Hybridization and washing steps were essentially performed as described by De Boer *et al* [87] with a hybridization step at 50°C for 16 hours. Samples were then blocked for 30 min at room temperature in PBS 1X containing 0.2% fish gelatin. Bound antisense cDNA probes were detected with a primary polyclonal sheep anti-digoxigenin antibody (Roche, 11333089001) used at 1:500 and a secondary Alexa 594-labeled donkey anti-sheep antibody (Molecular probes A11016, Life technologies) used at 1:1000. Control sense cDNA probes were used to confirm the specificity and the reliability of the signals observed with antisense probes

### Tissue localization of Hco-ACR-26 and Hco-ACR-27 subunits

Polyclonal antibodies were raised against synthetic peptide antigens designed from *Hco-acr-26* and *Hco-acr-27* deduced amino-acid sequences respectively (Eurogentec, Belgium). For Hco-ACR-26, the synthetic peptide “IPTEDHAGGRRYTDEQ” (position 13–28 from the predicted mature protein sequence) was injected into rabbits whereas for Hco-ACR-27, the synthetic peptide “RYDGKSPFTKDSFTE” (position 190–204) was injected into guinea pigs. Both antibodies were affinity purified and used for immunohistochemical staining on *H*. *contortus* L2 and adult stages. For *H*. *contortus* L2 stages, permeabilization was performed as described for *in-situ* hybridization. Permeabilized worms were fixed in -20°C methanol and -20°C acetone for 5 min each and washed twice in PBS 1X. Worms were then incubated with primary antibodies overnight at 4°C at the following dilutions in PBS 1X/ fish gelatin 0.1%/triton 0.25%: polyclonal rabbit anti-Hco-ACR-26, 1:500; polyclonal guinea pig anti-Hco-ACR-27, 1:500, monoclonal mouse anti-*C*. *elegans* myosin 5–6 heavy chain, 1:600 (DHSB). Samples were washed five times (20 min each) in washing solution (PBS 1X/triton 0.25%) and were incubated for 3 hours at room temperature with secondary antibodies at the following dilutions (in PBS 1X/ fish gelatin 0.1%/triton 0.25%): Alexa 594-labeled goat anti-rabbit IgG (H+L), 1:800 (Molecular bioprobes A11037, Life technologies); Alexa488-labeled goat anti-guinea pig IgG (H+L), 1:800 (Molecular bioprobes A11073, Life technologies); Alexa 350-labeled goat anti-mouse IgG (H+L), 1:1000 (Molecular bioprobes A21049, Life technologies) or Alexa 488-labeled goat anti-mouse IgG (H+L), 1:1000 (Molecular bioprobes A11001, Life technologies). After five washes of 20 min each in washing solution, worms were mounted on slides using the Vectashield mounting medium (Vector laboratories CA, USA) containing DAPI or not depending on the presence or absence of myosin staining respectively.

*H*. *contortus* adult males were embedded in OCT matrix (CellPath), frozen in isopentane cooled in liquid nitrogen, and sectioned using a microtome cryostat (Leica). Frozen 5 μm-thick sections were immunostained with a 1:200 dilution of rabbit anti-Hco-ACR-26, guinea pig anti-Hco-ACR-27 and mouse anti-myosin 5–6 antibodies (DSHB). After being washed in phosphate-buffered saline (PBS), sections were incubated with Alexa Fluor 594-conjugated goat anti-rabbit, Alexa Fluor 488-conjugated goat anti-guinea pig and Alexa Fluor 350-conjugated goat anti-mouse secondary antibodies at 1:200 dilutions. After further PBS washing, sections were mounted and visualized under a fluorescent confocal microscope. As negative controls, samples were incubated with secondary antibodies only or primary antibodies (anti-Hco-ACR-26 or anti-Hco-ACR-27) saturated with their respective peptide antigen (0.30mg/ml of each peptide). For each sample, immuno-histochemical staining experiments were performed at least 3 times independently to confirm the observations.

### Electrophysiology experiments

*H*. *contortus and P*. *equorum acr-26* and *acr-27* cDNAs were PCR-amplified using primers containing a restriction site in order to be sub-cloned into the expression vector pTB207 that is suitable for in vitro transcription [25]. Primers are listed in S3 Table. The resulting plasmids were linearized with the *NheI* restriction enzyme (Fermantas) and used as templates for cRNA synthesis using the T7 mMessage mMachine kit (Ambion). *Xenopus laevis* ovaries were obtained from the University of Rennes, France (CRB Xenope) and defolliculated using 2 mg/ml collagenase type II (Gibco) in the incubation solution containing: 100 mM NaCl, 2 mM KCl, 1.8 mM CaCl_2_.2H_2_O, 1 mM MgCl_2_.6H_2_O, 5 mM HEPES, 2.5 mM C_3_H_3_NaO_3_, pH 7.5, supplemented with penicillin 100 U/mL and streptomycin 100 μg/mL). The oocytes were injected in the animal pole with a total volume of 36 nL of cRNA mix containing 50 ng/μL of each cRNA in RNase-free water using the Drummond nanoject II microinjector. Microinjected oocytes were kept at 20°C in incubation medium for 4 days to allow the receptors expression. Two-electrode voltage-clamp recordings were carried out using an Oocyte Clamp OC-725C amplifier (Warner instrument) in oocytes being voltage-clamped at -60mV. All agonist preparations were prepared with recording buffer (100 mM NaCl, 2.5 mM KCl, 1 mM CaCl_2_.2H_2_O, 5 mM HEPES, pH 7.3). Currents were recorded and analyzed using the pCLAMP 10.4 package (Molecular Devices). EC_50_ values were determined using non-linear regression on normalized data (1 mM ACh as maximal response) using GraphPad Prism software.

### *Caenorhabditis elegans* experiments

Worms were maintained at 20°C on nematode growth medium (NGM) plates [89] and fed on a bacterial lawn (*Escherichia coli* OP50). Bristol N2 was used as a reference wild-type *C*. *elegans* strain. Dose-response assays were performed on gravid adults placed in wells of a 96-well plate containing M9 salt solution (3 g/L KH_2_PO_4_, 6 g/L Na_2_HPO_4_, 5 g/L NaCl, and 1 mM MgSO_4_ with BSA at 0.1%) with varying anthelmintic concentrations. After 10 min, worm thrashing was counted for 1 min. A thrash was defined as a change in the direction of bending at the mid-body to return to the initial position. For each drug concentration, 30 worms were tested. *Hco-acr-26*, *Hco-acr-27*, *Peq-acr-26* and *Peq-acr-27* coding sequences were sub-cloned into the pPD96.52 (Addgene) vector containing the muscle promoter *Pmyo3*. Primer sequences containing the suitable restriction sites are listed in S3 Table. Extrachromosomal transgenic strains of *C*. *elegans* were obtained by micro-injecting DNA into the gonads of young adult hermaphrodite worms as described [90]. N2 worms were injected with different mixes containing *Pmyo3*::*Hco-acr-26; Pmyo3*::*Hco-acr-27; Pmyo3*::*Peq-acr-26; Pmyo3*::*Peq-acr-27* alone or in combination (*Pmyo3*::*Hco-acr-26* + *Pmyo3*::*Hco-acr-27* or *Pmyo3*::*Peq-acr-26* + *Pmyo3*::*Peq-acr-27*) with a final concentration of 40 ng/μl for each plasmid. The plasmid pPD118.33_*Pmyo2*::*gfp* (Addgene) was co-injected at 25 ng/μl as a transformation marker. For each mixes, at least two independent lines were generated. For each line, thrashing assays were carried on at least 12 F1-worms carrying the selection marker. L4 larvae from N2 and transformed worms were transferred one day before the thrashing assay on NGM plate. One young adult was placed in each well of a 12-well plate containing M9 salt buffer. Thrashes produced by each worm during 1 minute were counted after a 10 minutes equilibration period. After drug application, thrashes were counted after 10, 20 and 30 minutes of exposure. Experimental data are shown as mean ± SEM. Statistical comparisons were done using the paired or unpaired Student’s t test or one-way ANOVA with Tukey’s multiple comparison test. A level of p<0.05 was considered significant. For each line, expression of the introduced *acr-26* and/or *acr-27* coding sequences from *H*. *contortus* or *P*. *equorum* was confirmed by RT-PCR experiments performed on 12 pooled worms. A partial sequence corresponding to the *C*. *elegans* endogenous AChR *unc-38* transcript was amplified with primers designed on two independent exons in order to control the cDNA template quality. Primer sequences are listed in S3 Table.

### Materials

Acetylcholine chloride (ACh), (-)-tetramisole hydrochloride (levamisole), (-)-nicotine hydrogen tartrate, pyrantel citrate, morantel citrate, oxantel pamoate, bephenium hydroxynaphthoate were purchased from Sigma-Aldrich.

### Accession numbers

The accession numbers sequences mentioned in this article are:

A***scaris suum*:** ACR-26 GU135625; ***Caenorhabditis elegans*:** ACR-2 NP_509128; ACR-3 NP_509129; ACR-5NP_498437; ACR-6 NP_491354; ACR-7 NP_495647; ACR-8 NP_509745; ACR-9 NP_510285; ACR-10 NP_508692; ACR-11 NP_491906; ACR-12 NP_510262; ACR-14 NP_495716; ACR-15 NP_505206; ACR-16 NP_505207; ACR-17 NP_001023961; ACR-18 NP_506868; ACR-19 NP_001129756; ACR-20 NP_001122627; ACR-21 NP_498452; ACR-23NP_504024; ACR-24 NP_001255866; ACR-25 NP_001023570; DEG-3 NP_505897; DES-2 NP_001256320; EAT-2 NP_496959; LEV-1 NP_001255705; LEV-8 NP_509932; UNC-29 NP_492399; UNC-38 NP_491472; UNC-63 NP_491533. ***Haemonchus contortus*:** actin DQ080917; ACR-26 EU006791; ACR-27 KC790461; β -tubulin isotype I EF198865; GAPDH HM145749; RIC3.1 HQ116823; UNC-74 HQ116821; UNC-50 HQ116822; ***Parascaris equorum*:** ACR-26 KP756902; ACR-27 KP756903. ***Aplysia californica*:** ACR-26-like AGM37744. ***Crassostrea gigas*:** ACR-26-like XP_011439437. ***Lottia gigantean*:** XP_009043985. ***Capitella teleta*:** ACR-26-like ELU14382. ***Daphnia pulex*:** ACR-26-like EFX90115. ***Ixodes scapularis*:** ACR-26-like AGV76072.

## Supporting Information

S1 Fig Maximum likelihood tree with bootstrap values showing relationships of ACR-26 and ACR-27 acetylcholine receptor (AChR) subunits from parasitic nematodes with *C*. *elegans* AChR subunits. Tree was built upon an alignment of AChR subunit sequences excluding the predicted signal peptide and the highly variable region between TM3 and TM4. Potential homologs of ACR-26 identified in the molluscs, *Aplysia californica*, *Crassostrea gigas*, *Lottia gigantean*, the annelid *Capitella telata* and the arthropods *Ixodes scapularis* and *Daphnia pulex* were also included in the analysis. The tree was rooted with DEG-3 group subunit sequences. Branch lengths are proportional to the number of substitutions per amino acid. Scale bar represents the number of substitution per site. The three letter prefixes in AChR subunit gene names, *Cel*, *Tci*, *Hco*, *Aca*, *Tca*, *Peq*, *Ovo*, *Dim*, *Llo*, *Bma*, *Sra and Ptr* refer to *Caenorhabditis elegans*, *Teladorsagia circumcincta*, *Haemonchus contortus*, *Ancylostoma caninum*, *Toxocara canis*, *Parascaris equorum*, *Onchocerca volvulus*, *Dirofilaria immitis*, *Loa loa*, *Brugia malayi*, *Strongyloides ratti* and *Parastrogyloides trichosuri* respectively.
(TIF)

S2 Fig Amino-acid alignments of ACR-26 and ACR-27 AChR subunit partial sequences from parasitic nematode species. Parasitic nematode species ACR-26 and ACR-27 partial amino-acid sequences (including the C- loop agonist binding site and the first transmembrane domain (TM1)) were aligned using the MUSCLE algorithm [36] and further processed using GeneDoc. Amino acids conserved between ACR-26 and ACR-27 sequences are highlighted in red. Amino acids specifically shared by ACR-26 homologs are highlighted in dark blue. Amino acids specifically shared by ACR-27 homologs are highlighted in light blue. The ACR-26 AChR subunits containing the prototypical cysteine doublet (YxCC) are defined as α-subunits (ACR-26) whereas the ACR-27 AChR subunits lacking this motif are defined as non-α-subunits. The three letter prefixes in AChR subunit gene names refer to: Aca: *Ancylostoma caninum;* Ace: *Ancylostoma ceylanicum;* Adu: *Ancylostoma duodenale;* Hbk: *Heligmosomoides bakeri;* Hco: *Haemonchus contortus;* Hpl: *Haemonchus placei;* Nam: *Necator americanus;* Nbr: *Nippostrongylus brasiliensis;* Tci: *Teladorsagia circumcincta;* Asu: *Ascaris suum;* Peq: *Parascaris equorum;* Tca: *Toxocara canis;* Bma: *Brugia malayi;* Dim: *Dirofilaria immitis;* Llo: *Loa loa;* Ovo: *Onchocerca volvulus;* Wba: *Wuchereria bancrofti;* Ptr: *Parastrongyloides trichosuri;* Spa: *Strongyloides papillosus;* Sra: *Strongyloides ratti;* Sst: *Strongyloides stercoralis;* Svz: *Strongyloides venezuelensis;* Bxy: *Bursaphelenchus xylophilus*.
(TIF)

S3 Fig Pharmacological profiles of Hco-L-AChR-1 and Hco-L-AChR-2. (A and B) Representative recording traces from a single oocyte expressing Hco-L-AChR-1 or Hco-L-AChR-2 challenged with 100 μM ACh and 100 μM of different anthelmintic compounds including levamisole (Lev), nicotine (Nic), pyrantel (Pyr) and morantel (Mor). The bars indicate the time period of the agonist application. Functional expression of Hco-L-AChR-1 requires the co-expression of Hco-UNC-38; Hco-UNC-63; Hco-UNC-29.1 and Hco-ACR-8 AChR subunits with the ancillary proteins Hco-RIC-3.1; Hco-UNC-50 and Hco-UNC-74 whereas Hco-L-AChR-2 requires the co-expression of Hco-UNC-38; Hco-UNC-63 and Hco-UNC-29.1 AChR subunits with the ancillary proteins Hco-RIC-3.1; Hco-UNC-50 and Hco-UNC-74. (C) Scatter plot (mean ± SEM) of normalized currents elicited by 100 μM Mor on Hco-26/27, Hco-L-AChR-1 and Hco-L-AChR-2. Currents have been normalized to and compared with 100 μM ACh elicited currents. Paired Student’s t-test, ***p<0.001. (D) Dose-response relationships of Hco-L-AChR-1 (black circle, n = 7) and Hco-L-AChR-2 (blue squares, n = 9) for Mor. Responses are normalized to 300 μM ACh elicited currents as described in Boulin *et al*. [25]. EC_50_ are 0.41±0.23 μM and 3.2±1.2 μM for Hco-L-AChR-1 and Hco-L-AChR-2 respectively.
(TIF)

S4 Fig Thrashing assays performed on *C*. *elegans* (N2) with different anthelmintic molecules. **(A)** Dose-response thrashing assays on wild-type *C*. *elegans* were performed using morantel (Mor; dark gray), pyrantel (Pyr; dotted) and levamisole (Lev; light gray). Thrashes of gravid wild-type adults were counted for one minute after 10 minutes of drug exposure. IC_50_ values for Mor, Pyr and Lev are 44±1.03 μM, 107.1±1.15 μM and 7±1.05 μM, respectively. Results are expressed as mean ± SEM derived from three independent sets of experiments. For each treatment, 30 worms were included in the analysis. **(B)** *C*. *elegans* thrashing assays in Mor (40 μM), Pyr (50 μM) and Lev (5 μM). Statistical analyses were performed using a One-Way Anova with Tukey’s Multiple Comparison Test with **p<0.01, ***p<0.001.
(TIF)

S5 Fig The co-expression of ACR-26 and ACR-27 from *H*. *contortus* or *P*. *equorum* in *C*. *elegans* increases its sensitivity to pyrantel (50 μM) but does not modulate its sensitivity to levamisole (5 μM). Thrashing assays were performed during 30 minutes with t0 corresponding to basal movements. For each *C*. *elegans* co-expressing ACR-26 and ACR-27 from *H*. *contortus* **(A and B)** or *P*. *equorum* **(C and D)**, two independent lines were used with >12 worms per lines. Thrashing assays performed with 50 μM Pyr or 5 μM Lev on N2 (WT) and transformed worms co-expressing ACR-26 and ACR-27 subunits. All results are expressed as mean ± SEM. Using *C*. *elegans* N2 as reference, statistical analysis was performed with an unpaired Student’s t-test with ***p<0.001, NS, not significant.
(TIF)

S1 Table Summary of *Hco-acr-26* and *Hco-acr-27* homologs identified in genomic databases from nematodes. Nematode clades as determined by Blaxter *et al*. [34]. Life style abbreviations: VP: vertebrate parasite; IP: Insect parasite; FL: free-living.; WBP: Worm BaseParasite. Database abbreviations: SI: Sanger Institute (www.sanger.ac.uk); NN: Nematode Net V4.0 (http://nematode.net/); WB: WormBase (www.wormbase.org/); *WBP*: WormBase Parasite *(* http://parasite.wormbase.org/).* complete or partial cDNA sequence available.
(DOCX)

S2 Table Ratio modulation of *Hco-acr-26* and *Hco-acr-27* cRNAs injected in *Xenopus* oocytes in combination with ancillary proteins. The relative maximal currents (Imax) have been normalized to those elicited by 100 μM acetylcholine on oocytes expressing Hco-ACR-26 and Hco-ACR-27 with different ratios. Responses from each oocyte were normalized to 100 μM acetylcholine. Results are shown as the mean ± SEM. ND: Not determined.* measurements not possible due to the small size of currents.
(DOCX)

S3 Table Sequences of primers used for amplifying *acr-26* and *acr-27* partial or complete coding sequences from *H*. *contortus* and *P*. *equorum*. Primers used for positive controls are also included.
(DOCX)
