# Supplementary figures and images for: Functional Characterization of a Novel Class of Morantel-Sensitive Acetylcholine Receptors in Nematodes
Source: PLoS Pathog. 2015 Dec 1;11(12):e1005267. doi: 10.1371/journal.ppat.1005267 (PMC4666645; doi:10.1371/journal.ppat.1005267)

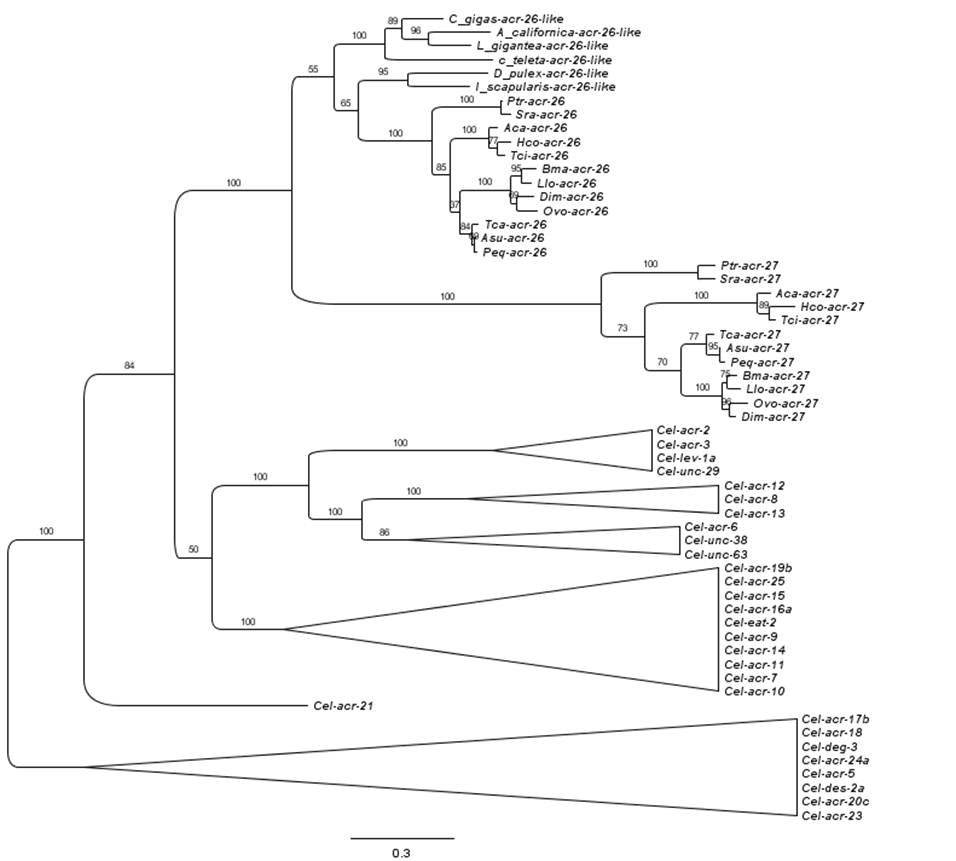

Supplement: S1 Fig — Tree was built upon an alignment of AChR subunit sequences excluding the predicted signal peptide and the highly variable region between TM3 and TM4. Potential homologs of ACR-26 identified in the molluscs, Aplysia californica, Crassostrea gigas, Lottia gigantean, the annelid Capitella telata and the arthropods Ixodes scapularis and Daphnia pulex were also included in the analysis. The tree was rooted with DEG-3 group subunit sequences. Branch lengths are proportional to the number of substitutions per amino acid. Scale bar represents the number of substitution per site. The three letter prefixes in AChR subunit gene names, Cel, Tci, Hco, Aca, Tca, Peq, Ovo, Dim, Llo, Bma, Sra and Ptr refer to Caenorhabditis elegans, Teladorsagia circumcincta, Haemonchus contortus, Ancylostoma caninum, Toxocara canis, Parascaris equorum, Onchocerca volvulus, Dirofilaria immitis, Loa loa, Brugia malayi, Strongyloides ratti and Parastrogyloides trichosuri respectively. (TIF) [file ppat.1005267.s001.tif]

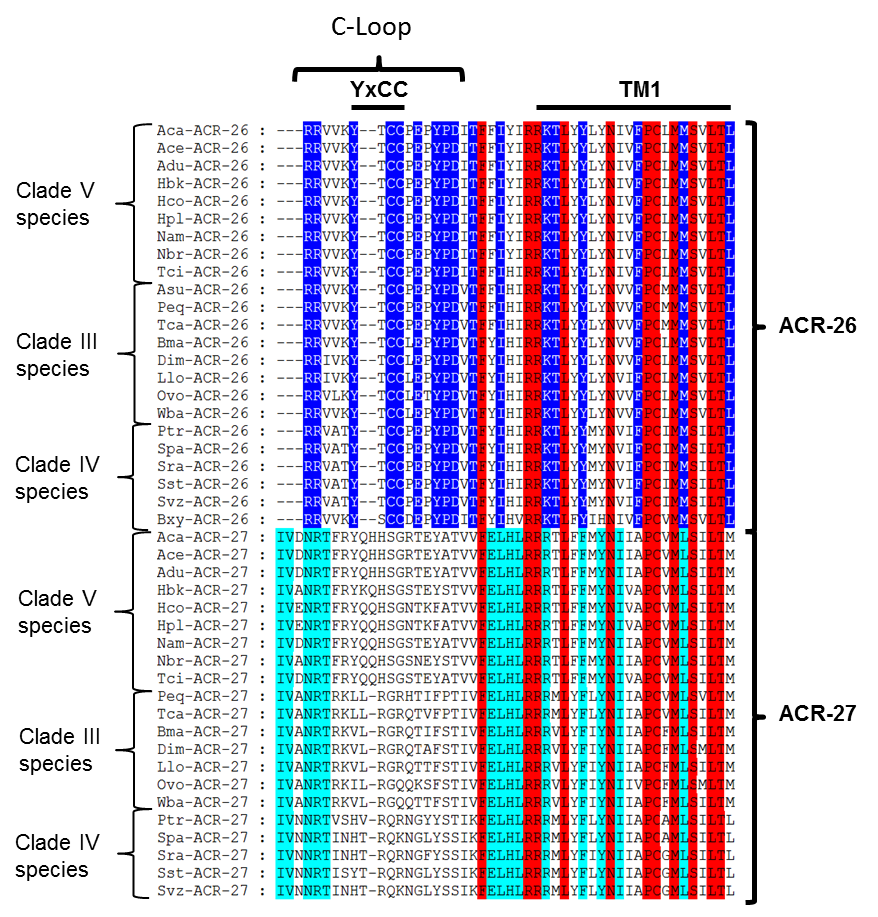

Supplement: S2 Fig — Parasitic nematode species ACR-26 and ACR-27 partial amino-acid sequences (including the C- loop agonist binding site and the first transmembrane domain (TM1)) were aligned using the MUSCLE algorithm [36] and further processed using GeneDoc. Amino acids conserved between ACR-26 and ACR-27 sequences are highlighted in red. Amino acids specifically shared by ACR-26 homologs are highlighted in dark blue. Amino acids specifically shared by ACR-27 homologs are highlighted in light blue. The ACR-26 AChR subunits containing the prototypical cysteine doublet (YxCC) are defined as α-subunits (ACR-26) whereas the ACR-27 AChR subunits lacking this motif are defined as non-α-subunits. The three letter prefixes in AChR subunit gene names refer to: Aca: Ancylostoma caninum; Ace: Ancylostoma ceylanicum; Adu: Ancylostoma duodenale; Hbk: Heligmosomoides bakeri; Hco: Haemonchus contortus; Hpl: Haemonchus placei; Nam: Necator americanus; Nbr: Nippostrongylus brasiliensis; Tci: Teladorsagia circumcincta; Asu: Ascaris suum; Peq: Parascaris equorum; Tca: Toxocara canis; Bma: Brugia malayi; Dim: Dirofilaria immitis; Llo: Loa loa; Ovo: Onchocerca volvulus; Wba: Wuchereria bancrofti; Ptr: Parastrongyloides trichosuri; Spa: Strongyloides papillosus; Sra: Strongyloides ratti; Sst: Strongyloides stercoralis; Svz: Strongyloides venezuelensis; Bxy: Bursaphelenchus xylophilus. (TIF) [file ppat.1005267.s002.tif]

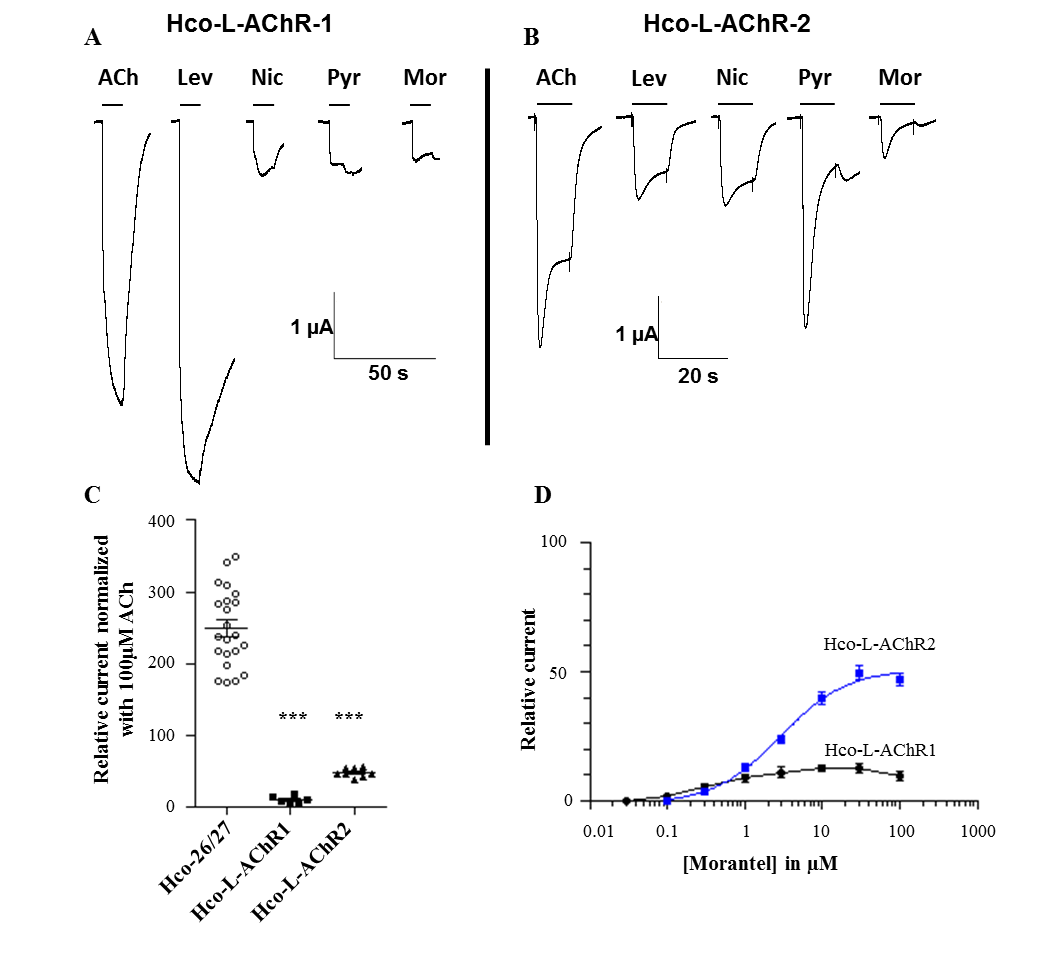

Supplement: S3 Fig — (A and B) Representative recording traces from a single oocyte expressing Hco-L-AChR-1 or Hco-L-AChR-2 challenged with 100 μM ACh and 100 μM of different anthelmintic compounds including levamisole (Lev), nicotine (Nic), pyrantel (Pyr) and morantel (Mor). The bars indicate the time period of the agonist application. Functional expression of Hco-L-AChR-1 requires the co-expression of Hco-UNC-38; Hco-UNC-63; Hco-UNC-29.1 and Hco-ACR-8 AChR subunits with the ancillary proteins Hco-RIC-3.1; Hco-UNC-50 and Hco-UNC-74 whereas Hco-L-AChR-2 requires the co-expression of Hco-UNC-38; Hco-UNC-63 and Hco-UNC-29.1 AChR subunits with the ancillary proteins Hco-RIC-3.1; Hco-UNC-50 and Hco-UNC-74. (C) Scatter plot (mean ± SEM) of normalized currents elicited by 100 μM Mor on Hco-26/27, Hco-L-AChR-1 and Hco-L-AChR-2. Currents have been normalized to and compared with 100 μM ACh elicited currents. Paired Student’s t-test, ***p<0.001. (D) Dose-response relationships of Hco-L-AChR-1 (black circle, n = 7) and Hco-L-AChR-2 (blue squares, n = 9) for Mor. Responses are normalized to 300 μM ACh elicited currents as described in Boulin et al. [25]. EC50 are 0.41±0.23 μM and 3.2±1.2 μM for Hco-L-AChR-1 and Hco-L-AChR-2 respectively. (TIF) [file ppat.1005267.s003.tif]

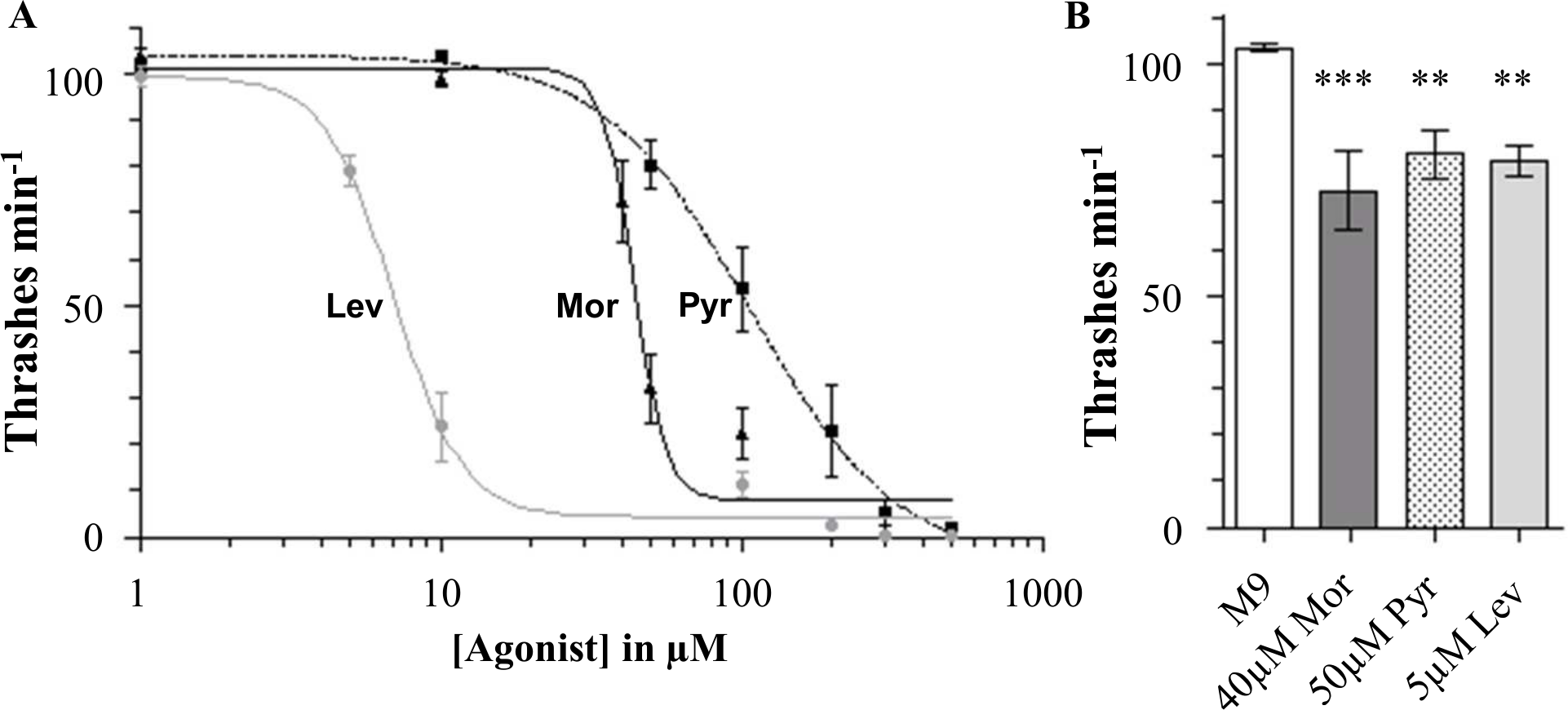

Supplement: S4 Fig — (A) Dose-response thrashing assays on wild-type C. elegans were performed using morantel (Mor; dark gray), pyrantel (Pyr; dotted) and levamisole (Lev; light gray). Thrashes of gravid wild-type adults were counted for one minute after 10 minutes of drug exposure. IC50 values for Mor, Pyr and Lev are 44±1.03 μM, 107.1±1.15 μM and 7±1.05 μM, respectively. Results are expressed as mean ± SEM derived from three independent sets of experiments. For each treatment, 30 worms were included in the analysis. (B) C. elegans thrashing assays in Mor (40 μM), Pyr (50 μM) and Lev (5 μM). Statistical analyses were performed using a One-Way Anova with Tukey’s Multiple Comparison Test with **p<0.01, ***p<0.001. (TIF) [file ppat.1005267.s004.tif]

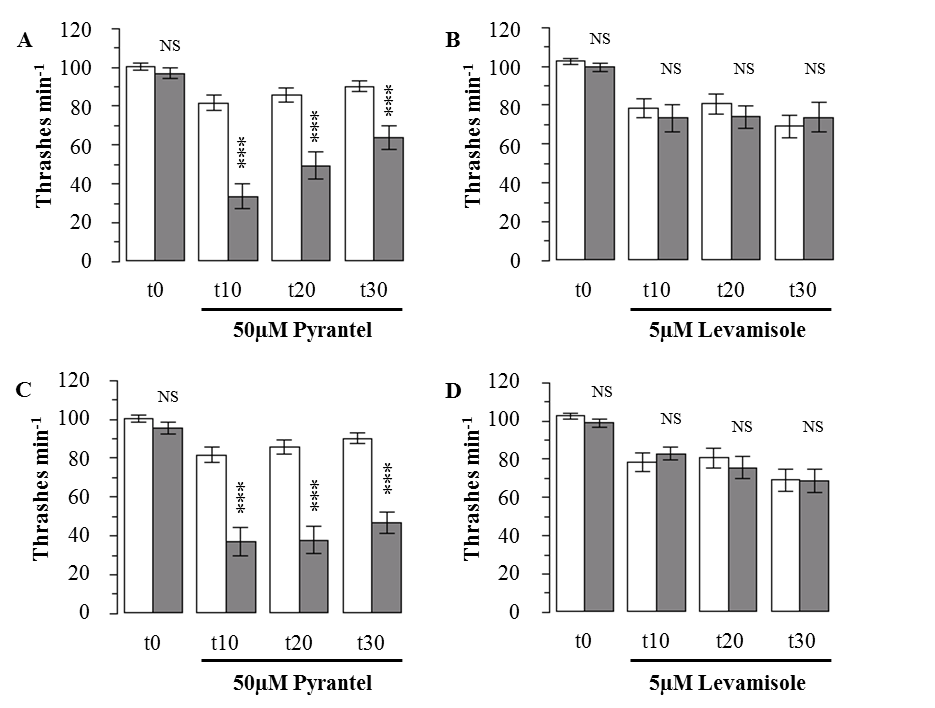

Supplement: S5 Fig — Thrashing assays were performed during 30 minutes with t0 corresponding to basal movements. For each C. elegans co-expressing ACR-26 and ACR-27 from H. contortus (A and B) or P. equorum (C and D), two independent lines were used with >12 worms per lines. Thrashing assays performed with 50 μM Pyr or 5 μM Lev on N2 (WT) and transformed worms co-expressing ACR-26 and ACR-27 subunits. All results are expressed as mean ± SEM. Using C. elegans N2 as reference, statistical analysis was performed with an unpaired Student’s t-test with ***p<0.001, NS, not significant. (TIF) [file ppat.1005267.s005.tif]
