# Supplementary material for: Functional Characterization of a Novel Class of Morantel-Sensitive Acetylcholine Receptors in Nematodes
Source: PLoS Pathog. 2015 Dec 1;11(12):e1005267. doi: 10.1371/journal.ppat.1005267 (PMC4666645; doi:10.1371/journal.ppat.1005267)
Supplement: S1 Table — Nematode clades as determined by Blaxter et al. [34]. Life style abbreviations: VP: vertebrate parasite; IP: Insect parasite; FL: free-living.; WBP: Worm BaseParasite. Database abbreviations: SI: Sanger Institute (www.sanger.ac.uk); NN: Nematode Net V4.0 (http://nematode.net/); WB: WormBase (www.wormbase.org/); WBP: WormBase Parasite ( http://parasite.wormbase.org/).* complete or partial cDNA sequence available. (DOCX) [file ppat.1005267.s006.docx]

|  | **Nematode species** | **Life- style** | **Database** | **ACR**  **26** | **sequence reference** | **Coding sequence length (bp)** | **ACR**  **27** | **sequence reference** | **Coding sequence length (bp)** |
| --- | --- | --- | --- | --- | --- | --- | --- | --- | --- |
| **CLADE I** | ***Romanomermis culixivorax*** | **IP** | **WBP (PRJEB1358)** | **NO** |  |  | **NO** |  |  |
|  | ***Soboliphyme baturini*** | **VP** | **WBP (PRJEB516)** | **NO** |  |  | **NO** |  |  |
|  | ***Trichinella nativa*** | **VP** | **WBP (PRJNA179527)** | **NO** |  |  | **NO** |  |  |
|  | ***Trichinella spiralis*** | **VP** | **WBP (PRJNA12603)** | **NO** |  |  | **NO** |  |  |
|  | ***Trichuris muris*** | **VP** | **WBP (PRJEB126)** | **NO** |  |  | **NO** |  |  |
|  | ***Trichuris suis*** | **VP** | **WBP (PRJNA179528)** | **NO** |  |  | **NO** |  |  |
|  | ***Trichuris trichiura*** | **VP** | **WBP (PRJEB535)** | **NO** |  |  | **NO** |  |  |
|  | ***Xiphinema index*** | **PP** | **NN** | **NO** |  |  | **NO** |  |  |
| **CLADE III** | ***Acanthocheilonema viteae*** | **VP** | **WBP (PRJEB4306)** | **YES** | **nAV.1.0 scaffold 00026** | **1035** | **YES** | **nAV.1.0 scaffold 00332** | **1239** |
|  | ***Anisakis simplex*** | **VP** | **WBP (PRJEB496)** | **YES** | **ASIM scaffold 0000040** | **882** | **YES** | **ASIM scaffold 0001337** | **1335** |
|  | ***Ascaris lumbricoides*** | **VP** | **WBP (PRJEB495)** | **YES** | **ALUE scaffold 0000918** | **1509** | **YES** | **ALUE scaffold 0001837** | **1320** |
|  | ***Ascaris suum*** | **VP** | **GB** | **YES*** | **GU135625 [28]** | **1533** | **YES** | **Scaffold 5** | **1293** |
|  | ***Brugia malayi*** | **VP** | **WBP (PRJNA10729)** | **YES** | **Bmal v3 scaffold 1** | **1476** | **YES** | **Bmal v3 scaffold 111** | **1443** |
|  | ***Brugia pahangi*** | **VP** | **WBP (PRJEB497)** | **YES** | **BPAG contig 0000045** | **1254** | **YES** | **BPAG scaffold 0000221** | **1017** |
|  | ***Brugia timori*** | **VP** | **WBP (PRJEB4663)** | **YES** | **BTMF scaffold 0001466** | **1449** | **YES** | **BTMF scaffold 0005518**  **BTMF scaffold 0014713**  **BTMF scaffold 0002515** | **1416** |
|  | ***Dirofilaria immitis*** | **VP** | **WBP (PRJEB1797)** | **YES** | **nDi.2.2.scaffold 00029** | **1476** | **YES** | **nDi.2.2.scaffold 00111** | **1443** |
|  | ***Dracunculus medinensis*** | **VP** | **WBP (PRJEB500)** | **YES** | **DME scaffold 0000016** | **1263** | **YES** | **DME scaffold 0000002** | **1257** |
|  | ***Elaeophora elaphi*** | **VP** | **WBP (PRJEB502)** | **YES** | **EEL scaffold 0000005** | **1491** | **YES** | **EEL contig 0000176** | **1461** |
|  | ***Enterobius vermicularis*** | **VP** | **WBP (PRJEB503)** | **NO** |  |  | **NO** |  |  |
|  | ***Gongylonema pulchrum*** | **VP** | **SI (Gpu contigs V1)** | **YES** | **GPUH.contig.51721.1736 GPUH.contig.45747.1998 GPUH.contig.128127.504** | **1188** | **YES** | **GPUH.contig.10432.6582**  **GPUH.contig.32593.2834**  **GPUH.contig.19313.4391**  **GPUH.contig.61666.1399**  **GPUH.contig.66291.1273** | **879** |
|  | ***Litomosoides sigmodontis*** | **VP** | **WB (PPRJEB3075)** | **NO** |  |  | **NO** |  |  |
|  | ***Loa loa*** | **VP** | **WB (PPRJNA60051)** | **YES** | **JH712085** | **1500** | **YES** | **JH712555** | **1434** |
|  | ***Onchocerca flexuosa*** | **VP** | **SI (Ofl contigs V1)** | **YES** | **OFLC contig 05909.3253 OFLC contig 19328.1206 OFLC contig 35625.661 OFLC scaffold00489.13246** | **1326** | **YES** | **OFLC contig 11963.1845**  **OFLC contig 11340.1930**  **OFLC.contig.17381.1329** | **1116** |
|  | ***Onchocerca ochengi*** | **VP** | **WB (PPRJEB1809)** | **YES** | **nOo.2.0.Scaffold 13615 nOo.2.0.Scaffold 01417** | **1137** | **YES** | **nOo.2.0.Scaffold 05925**  **nOo.2.0.Scaffold 11562** | **1050** |
|  | ***Onchocerca volvulus*** | **VP** | **WB (PPRJEB513)** | **YES** | **OVOC_OM1b supercontig** | **1491** | **YES** | **OVOC_OM3 supercontig** | **1440** |
|  | ***Parascaris equorum*** | **VP** | **GB** | **YES*** | **KP756902** | **1521** | **YES*** | **KP756903** | **1350** |
|  | ***Syphacia muris*** | **VP** | **WBP(PRJEB524)** | **NO** |  |  | **NO** |  |  |
|  | ***Toxocara canis*** | **VP** | **WBP(PRJEB533)** | **YES** | **TCNE scaffold 0001835** | **1392** | **YES** | **TCNE scaffold00230.95622** | **1293** |
|  | ***Thelazia callipaeda*** | **VP** | **SI (Tzc contigs V1)** | **YES** | **TCLT scaffold 00028.166293** | **1515** | **YES** | **TCLT scaffold 00324.60166** | **1398** |
|  | ***Wuchereria bancrofti*** | **VP** | **SI (Wba contigs V2)** | **YES** | **WBA_000032** | **1479** | **YES** | **WBA_001016**  **WBA_004443** | **1443** |
| **CLADE IV** | ***Bursaphelenchus xylophilus*** | **PP** | **WBP(PRJEA64437)** | **YES** | **BUX.scaffold01198** | **1503** | **NO** |  |  |
|  | ***Ditylenchus africanus*** | **PP** | **NN** | **NO** |  |  | **NO** |  |  |
|  | ***Globodera pallida*** | **PP** | **WBP (PRJEB123)** | **NO** |  |  | **NO** |  |  |
|  | ***Globodera rostochiensis*** | **PP** | **NN** | **NO** |  |  | **NO** |  |  |
|  | ***Heterodera glycines*** | **PP** | **NN** | **NO** |  |  | **NO** |  |  |
|  | ***Heterodera shachtii*** | **PP** | **NN** | **NO** |  |  | **NO** |  |  |
|  | ***Laxus oneistus*** | **FL** | **NN** | **NO** |  |  | **NO** |  |  |
|  | ***Meloidogyne arenaria*** | **PP** | **NN** | **NO** |  |  | **NO** |  |  |
|  | ***Meloidogyne chitwoodi*** | **PP** | **NN** | **NO** |  |  | **NO** |  |  |
|  | ***Meloidogyne floridensis*** | **pp** | **WBP (PRJEB6016)** | **NO** |  |  | **NO** |  |  |
|  | ***Meloidogyne hapla*** | **PP** | **WBP (PRJNA29083)** | **NO** |  |  | **NO** |  |  |
|  | ***Meloidogyne incognita*** | **PP** | **WBP (PRJEA28837)** | **NO** |  |  | **NO** |  |  |
|  | ***Meloidogyne javanica*** | **PP** | **NN** | **NO** |  |  | **NO** |  |  |
|  | ***Meloidogyne paranaensis*** | **PP** | **NN** | **NO** |  |  | **NO** |  |  |
|  | ***Panagrellus redivivus*** | **FL** | **WB PRJNA186477** | **YES** | **KB455485** | **1668** | **YES** | **KB455153** | **1389** |
|  | ***Parastrongyloides trichosuri*** | **VP** | **WBP PRJEB515** | **YES** | **PTRK scaffold15** | **1557** | **YES** | **PTRK scaffold0000005** | **1368** |
|  | ***Pratylenchus penetrans*** | **PP** | **NN** | **NO** |  |  | **NO** |  |  |
|  | ***Pratylenchus vulnus*** | **PP** | **NN** | **NO** |  |  | **NO** |  |  |
|  | ***Rhabditophanes sp.kr3021*** | **FL** | **WBP (PRJEB1297)** | **YES** | **RSKR contig 0000025** | **1515** | **YES** | **RSKR contig 0000009** | **1347** |
|  | ***Steinernema carpocapsae*** | **IP** | **WBP (PRJNA202318)** | **NO** |  |  | **NO** |  |  |
|  | ***Steinernema feltiae*** | **IP** | **WBP (PRJNA204661)** | **YES** | **FELT scaffold 3761** | **1293** | **YES** | **FELT scaffold 4267** | **1164** |
|  | ***Steinernema glaseri*** | **IP** | **WBP (PRJNA204943)** | **YES** | **GLAS 2882 dna:scaffold** | **1329** | **YES** | **GLAS 6236 dna:scaffold** | **1497** |
|  | ***Steinernema monticolum*** | **IP** | **WBP (PRJNA205067)** | **YES** | **MONTI 11882 dna:scaffold** | **1335** | **YES** | **MONTI 3801 dna:scaffold** | **1143** |
|  | ***Steinernema scapterisci*** | **IP** | **WBP (PRJNA204942)** | **NO** |  |  | **NO** |  |  |
|  | ***Strongyloides papillosus*** | **VP** | **WBP (PRJEB525)** | **YES** | **SPAL_scaffold 11** | **1545** | **YES** | **SPAL_scaffold 24** | **1422** |
|  | ***Strongyloides ratti*** | **VP** | **WBP (PRJEB125)** | **YES** | **SRAE Chr1 scaffold 1** | **1545** | **YES** | **SRAE Chr2 scaffold 1** | **1422** |
|  | ***Strongyloides stercoralis*** | **VP** | **WBP (PRJEB528)** | **YES** | **SSTP contig 0000022** | **1545** | **YES** | **SSTP contig 0000045** | **1479** |
|  | ***Strongyloides venezuelensis*** | **VP** | **WBP (PRJEB530)** | **YES** | **SVE scaffold 0000003** | **1545** | **YES** | **SVE scaffold 0000001** | **1422** |
|  | ***Radopholus similis*** | **PP** | **NN** | **NO** |  |  | **NO** |  |  |
|  | ***Zeldia punctata*** | **FL** | **NN** | **NO** |  |  | **NO** |  |  |
| **CLADE V** | ***Ancylostoma caninum*** | **VP** | **WBP (PRJNA72585)** | **YES** | **ANCCANDFT contig 381** | **1575** | **YES** | **ANCCANDFT contig 279** | **1443** |
|  | ***Ancylostoma ceylanicum*** | **VP** | **WBP (PRJNA231479)** | **YES** | **Acey s0011** | **1575** | **YES** | **Acey s0724** | **1443** |
|  | ***Ancylostoma duodenale*** | **VP** | **WBP (PRJNA72581)** | **YES** | **ANCDUODFT contig 13513** | **1554** | **YES** | **ANCDUODFT contig 5556** | **1449** |
|  | ***Angiostrongylus cantonensis*** | **VP** | **WBP (PRJEB493)** | **NO** |  |  | **NO** |  |  |
|  | ***Angiostrongylus costaricensis*** | **VP** | **WBP (PRJEB494)** | **NO** |  |  | **NO** |  |  |
|  | ***Caenorhabditis angaria*** | **FL** | **WB (PRJNA51225)** | **NO** |  |  | **NO** |  |  |
|  | ***Caenorhabditis brenneri*** | **FL** | **WB (PRJNA20035)** | **NO** |  |  | **NO** |  |  |
|  | ***Caenorhabditis briggsae*** | **FL** | **WB (PRJNA10731)** | **NO** |  |  | **NO** |  |  |
|  | ***Caenorhabditis elegans*** | **FL** | **WB (PRJNA13758)** | **NO** |  |  | **NO** |  |  |
|  | ***Caenorhabditis japonica*** | **FL** | **WB (PRJNA12591)** | **NO** |  |  | **NO** |  |  |
|  | ***Caenorhabditis remanei*** | **FL** | **WB (PRJNA53967)** | **NO** |  |  | **NO** |  |  |
|  | ***Caenorhabditis sp5*** | **FL** | **WB (PRJNA194557)** | **NO** |  |  | **NO** |  |  |
|  | ***Caenorhabditis tropicalis*** | **FL** | **WB (PRJNA53597)** | **NO** |  |  | **NO** |  |  |
|  | ***Cooperia oncophora*** | **VP** | **GB/NN** | **YES** | **JN966889 [28]** | **639** | **YES** | **Conc contig 21153** | **1377** |
|  | ***Cylicostephanus goldi*** | **VP** | **WBP (PRJEB498)** | **YES** | **CGOC scaffold 0025384 CGOC scaffold 0046683 CGOC contig 0081091** | **621** | **YES** | **CGOC scaffold 0008233**  **CGOC contig 0024838**  **CGOC contig 0102297**  **CGOC contig 36258** | **882** |
|  | ***Dictyocaulus_viviparus*** | **VP** | **WBP (PRJEB5116)** | **NO** |  |  | **NO** |  |  |
|  | ***Heterorhabditis bacteriophora*** | **IP** | **WBP (PRJNA13977)** | **YES** | **scaffold 59** | **1380** | **NO** |  |  |
|  | ***Haemonchus contortus*** | **VP** | **GB** | **YES*** | **EU006791 [28]** | **GB** | **YES*** | **KC790461** |  |
|  | ***Haemonchus placei*** | **VP** | **WBP (PRJEB509)** | **YES** | **HPLM scaffold 0005539** | **1560** | **YES** | **HPLM scaffold 0000825**  **GB (LM583880.1)** | **1446** |
|  | ***Heligmosomoides bakeri*** | **VP** | **WBP (PRJEB1203)** | **YES** | **HPBE scaffold 0002644** | **1560** | **YES** | **HPBE scaffold 0000168**  **HPBE scaffold 0013412**  **HPBE contig 0002410** | **1452** |
|  | ***Necator americanus*** | **VP** | **WBP (PRJNA72135)** | **YES** | **KI660223** | **1566** | **YES** | **KI658002** | **1143** |
|  | ***Nippostrongylus brasiliensis*** | **VP** | **WBP (PRJEB511)** | **YES** | **NBR scaffold 0000066** | **1593** | **YES** | **NBR scaffold 0000558** | **1446** |
|  | ***Oesophagostomum dentatum*** | **VP** | **WBP (PRJNA72579)** | **YES** | **OESDENDFT contig 688** | **1203** | **YES** | **OESDENDFT contig 17100**  **OESDENDFT contig 757649**  **OESDENDFT contig 42226**  **OESDENDFT contig 732480** | **618** |
|  | ***Ostertagia ostertagi*** | **VP** | **NN/GB** | **YES** | **isotig. 25400 / JN 966890** | **717** | **YES** | **Isotig. 23185** | **585** |
|  | ***Pristionchus expectatus*** | **FL** | **WBP (PRJEB6009)** | **NO** |  |  | **NO** |  |  |
|  | ***Pristionchus pacificus*** | **FL** | **WBP (PRJNA12644)** | **NO** |  |  | **NO** |  |  |
|  | ***Strongylus vulgaris*** | **VP** | **SI (Svu contigs v1)** | **YES** | **SVUK contig 10771.4516 SVUK contig 141869.647 SVUK contig 157157.557 SVUK contig 116910.790 SVUK contig 78389.1163** | **987** | **YES** | **SVUK scaffold. 21102.3081**  **SVUK contig 21699.3032** | **1221** |
|  | ***Teladorsagia circumcincta*** | **VP** | **WBP (PRJNA72569)** | **YES** | **TELCIRDFT contig 625** | **1557** | **YES** | **TELCIRDFT contig24** | **1443** |
|  | ***Trichostrongylus colubriformis*** | **VP** | **NN** | **NO** |  |  | **NO** |  |  |
