# Supplementary material for: Functional Characterization of a Novel Class of Morantel-Sensitive Acetylcholine Receptors in Nematodes
Source: PLoS Pathog. 2015 Dec 1;11(12):e1005267. doi: 10.1371/journal.ppat.1005267 (PMC4666645; doi:10.1371/journal.ppat.1005267)
Supplement: S2 Table — The relative maximal currents (Imax) have been normalized to those elicited by 100 μM acetylcholine on oocytes expressing Hco-ACR-26 and Hco-ACR-27 with different ratios. Responses from each oocyte were normalized to 100 μM acetylcholine. Results are shown as the mean ± SEM. ND: Not determined.* measurements not possible due to the small size of currents. (DOCX) [file ppat.1005267.s007.docx]

| **Agonists** | **1:1**  **26/27** | **1:5**  **26/27** | **5:1**  **26/27** |
| --- | --- | --- | --- |
| Acetylcholine (Imax) | 100 | 100 | 100 |
| Pyrantel (Imax) | 81.01±6.21 | 83.12±17.92 | 84.13±8.54 |
| Morantel (Imax) | 249.20±11.50 | 137.0±19.66 | 264.9±29.12 |
| Oxantel (Imax) | 0.95±0.95 | * | * |
| Nicotine (Imax) | 1.25±0.94 | 6.1±3.98 | 1.59±1.59 |
| Levamisole (Imax) | 3.46±1.44 | 4.18±2.89 | 1.77±1.77 |
| Bephnium (Imax) | 3.21±1.61 | 2.26±2.26 | * |
