# Supplementary material for: Functional Characterization of a Novel Class of Morantel-Sensitive Acetylcholine Receptors in Nematodes
Source: PLoS Pathog. 2015 Dec 1;11(12):e1005267. doi: 10.1371/journal.ppat.1005267 (PMC4666645; doi:10.1371/journal.ppat.1005267)
Supplement: S3 Table — Primers used for positive controls are also included. (DOCX) [file ppat.1005267.s008.docx]

|  | Primer name | Primer sequence |
| --- | --- | --- |
| 5’ and 3’ cDNA ends  identification | *Hco-acr-27-F1* | ATGCCGTCTAGACGTAGGTCA |
|  | *Hco-acr-27-F2* | TAGCTCGTTGGTCACAATTGG |
|  | *Hco-acr-27-R1* | CCAATTGTGACCAACGAGCTA |
|  | *Hco-acr-27-R2* | CTCAGCTCCAACTCGATCAAT |
|  | *Peq-acr-26-F1* | TGGCGACAAGTGGCCCAAGTG |
|  | *Peq-acr-26-F2* | ATCCGATGTGTTCGACGAGTC |
|  | *Peq-acr-27-F1* | ATGCTGCCATGTGAGAGTGGC |
|  | *Peq-acr-27-F2* | TCGGATTGACGGTACTTCTCG |
|  | *Peq-acr-26-R1* | GACTCGTCGAACACATCGGAT |
|  | *Peq-acr-26-R2* | CACTTGGGCCACTTGTCGCCA |
|  | *Peq-acr-27-R1* | CGAGAAGTACCGTCAATCCGA |
|  | *Peq-acr-27-R2* | GCCACTCTCACATGGCAGCAT |
| Complete coding sequence  amplification | *Hco-acr-26-Fcomp* | ATGTTGATCTTCTGCTCAATT |
|  | *Hco-acr-26-Rcomp* | CTACTCCGGGTCCTCATAATT |
|  | *Hco-acr-27-Fcomp* | GGCCTCGGCTTCAGTCATGTA |
|  | *Hco-acr-27-Rcomp* | TCACAATTCCCACACTCGATC |
|  | *Peq-acr-26-Fcomp* | ATGATGGCAACTCGTCGGCGT |
|  | *Peq-acr-26-Rcomp* | AAAACTTTAATGCAGACCATA |
|  | *Peq-acr-27-Fcomp* | CTCTACTCATGCTCTGCATCA |
|  | *Peq-acr-27-Rcomp* | TCAAGAGTCATCATGCGAATCA |
| RT-PCR &  Quantitative-RT-PCR  Performed on *H. contortus* cDNA | *Hco-acr-26-RTF* | TACGGAAACGACCACGAGTT |
|  | *Hco-acr-26-RTR* | ATGGTTGCGGTCGCGAACA |
|  | *Hco-acr-27-RTF* | TATGCCGTCTAGACGTAGGT |
|  | *Hco-acr-27-RTR* | ACAGTGAAGAATAGACGATCGA |
|  | *Hco-actin-RTF* | ACAGGATGCAGAAAGAAATCAC |
|  | *Hco-actin-RTR* | TGGACAGAGAGGCAAGGATAG |
|  | *Hco-gapdh-RTF* | GTGTGAACCACGAGACCTACA |
|  | *Hco-gapdh-RTR* | TATCGTCCATGCTAGCTGGTT |
|  | *Hco-Btub-RTF* | CCAATTGACGCATTCACTTG |
|  | *Hco-Btub-RTR* | GATCAGCATTCAGCTGTCCA |
| *in-situ*  hybridiz-ation | *Hco-acr-26 HISF* | **ACTGGTGGGAATGGAGTCGCT** |
|  | *Hco-acr-26-HISR* | CTACTCCGGGTCCTCATAATT |
|  | *Hco-acr-27 HISF* | ATTTACGTCACGGAGCCGATT |
|  | *Hco-acr-27-HISR* | TCACAATTCCCACACTCGATC |
| pTB207  subcloning | *Hco-acr-26-FHind3* | aaaaagcttATGTTGATCTTCTGCTCAATT |
|  | *Hco-acr-26-RNot1* | tttgcggccgcCTACTCCGGGTCCTCATAATT |
|  | *Hco-acr-27-FHind3* | aaaaagcttGGCCTCGGCTTCAGTCATGTA |
|  | *Hco-acr-27-RNot1* | tttgcggccgcTCACAATTCCCACACTCGATC |
|  | *Peq-acr-26-FNot1* | tttgcggccgcATGATGGCAACTCGTCGGCGT |
|  | *Peq-acr-26-RNot1* | tttgcggccgcAAAACTTTAATGCAGACCATA |
|  | *Peq-acr-27-FNot1* | tttgcggccgcCTCTACTCATGCTCTGCATCA |
|  | *Peq-acr-27-RApa1* | tttttgggcccTCAAGAGTCATCATGCGAATCA |
| ppD96.52 subcloning | *Hco-acr-26-FBamH1* | tttttggatccATGTTGATCTTCTGCTCAATTAT |
|  | *Hco-acr-26-RKpn1* | tttttggtaccCATACTGTATCTACTCCGGGT |
|  | *Hco-acr-27-FBamH1* | tttttggatccGCCTCGGCTTCAGTCATGTA |
|  | *Hco-acr-27-RKpn1* | tttttggtaccTCACAATTCCCACACTCGATC |
|  | *Peq-acr-26-FBamH1* | tttttggatccATGATGGCAACTCGTCGGCGT |
|  | *Peq-acr-26-RKpn1* | tttttggtaccAAAACTTTAATGCAGACCATA |
|  | *Peq-acr-27-FBamH1* | tttttggatccCTCTACTCATGCTCTGCATCA |
|  | *Peq-acr-27-RKpn1* | tttttggtaccTCAAGAGTCATCATGCGAATCA |
| RT-PCR Performed on transgenic *C. elegans* | *Cel-unc-38-F* | ACCAAGTCTCCGATACGAAGA |
|  | *Cel-unc-38-R* | TTGACTGTGTAGAAGAGCGGT |
|  | *Hco-acr-26-RTF* | TACGGAAACGACCACGAGTT |
|  | *Hco-acr-26-RTR* | ATGGTTGCGGTCGCGAACA |
|  | *Hco-acr-27-RTF* | TATGCCGTCTAGACGTAGGT |
|  | *Hco-acr-27-RTR* | ACAGTGAAGAATAGACGATCGA |
|  | *Peq-acr-26-RTF* | AGTACTGGGCTTGCTATGGAT |
|  | *Peq-acr-26-RTR* | ATTGAGCGATGCACTGTAGGT |
|  | *Peq-acr-27-RTF* | GTGAGAGTGGCGAGAAAGTGA |
|  | *Peq-acr-27-RTR* | TATTATCGCGAGCTGACTCCA |
